# Molecular Pathways and Roles for Vitamin K2-7 as a Health-Beneficial Nutraceutical: Challenges and Opportunities

**DOI:** 10.3389/fphar.2022.896920

**Published:** 2022-06-14

**Authors:** Nikita Jadhav, Saiprasad Ajgaonkar, Praful Saha, Pranay Gurav, Amitkumar Pandey, Vivek Basudkar, Yash Gada, Sangita Panda, Shashank Jadhav, Dilip Mehta, Sujit Nair

**Affiliations:** ^1^ Viridis Biopharma Pvt. Ltd., Mumbai, India; ^2^ Synergia Life Sciences Pvt. Ltd., Mumbai, India

**Keywords:** vitamin K2-7, menaquinone, clinical trial, nutraceutical, osteocalcin, diabetes, neuropathy, cancer

## Abstract

Vitamin K2-7, also known as menaquinone-7 (MK-7) is a form of vitamin K that has health-beneficial effects in osteoporosis, cardiovascular disease, inflammation, cancer, Alzheimer’s disease, diabetes and peripheral neuropathy. Compared to vitamin K1 (phylloquinone), K2-7 is absorbed more readily and is more bioavailable. Clinical studies have unequivocally demonstrated the utility of vitamin K2-7 supplementation in ameliorating peripheral neuropathy, reducing bone fracture risk and improving cardiovascular health. We examine how undercarboxylated osteocalcin (ucOC) and matrix Gla protein (ucMGP) are converted to carboxylated forms (cOC and cMGP respectively) by K2-7 acting as a cofactor, thus facilitating the deposition of calcium in bones and preventing vascular calcification. K2-7 is beneficial in managing bone loss because it upregulates osteoprotegerin which is a decoy receptor for RANK ligand (RANKL) thus inhibiting bone resorption. We also review the evidence for the health-beneficial outcomes of K2-7 in diabetes, peripheral neuropathy and Alzheimer’s disease. In addition, we discuss the K2-7-mediated suppression of growth in cancer cells *via* cell-cycle arrest, autophagy and apoptosis. The mechanistic basis for the disease-modulating effects of K2-7 is mediated through various signal transduction pathways such as PI3K/AKT, MAP Kinase, JAK/STAT, NF-κB, *etc*. Interestingly, K2-7 is also responsible for suppression of proinflammatory mediators such as IL-1α, IL-1β and TNF-α. We elucidate various genes modulated by K2-7 as well as the clinical pharmacometrics of vitamin K2-7 including K2-7-mediated pharmacokinetics/pharmacodynamics (PK/PD). Further, we discuss the current status of clinical trials on K2-7 that shed light on dosing strategies for maximum health benefits. Taken together, this is a synthetic review that delineates the health-beneficial effects of K2-7 in a clinical setting, highlights the molecular basis for these effects, elucidates the clinical pharmacokinetics of K2-7, and underscores the need for K2-7 supplementation in the global diet.

## Introduction

### Chemical Structure of Vitamin K

**FIGURE 1:**
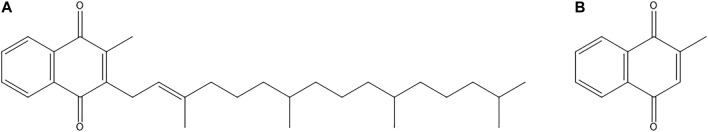
Chemical structures of **(A)** Phylloquinone (vitamin K1); and **(B)** Menadione (vitamin K3).

**FIGURE 2:**
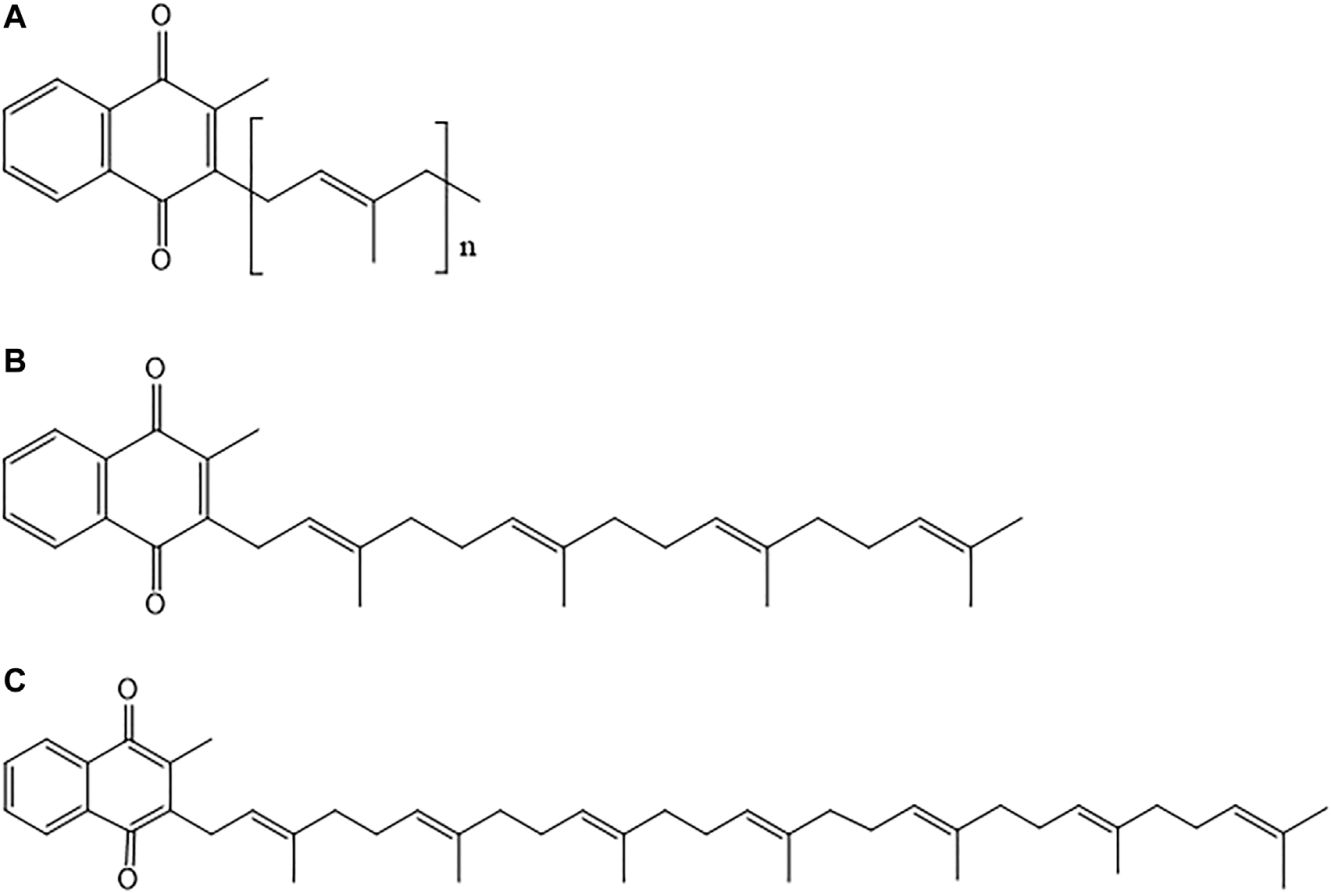
**(A)** General chemical structure of vitamin K2 family; **(B)** Chemical structure of menaquinone-4 (MK-4); and **(C)** Chemical structure of menaquinone-7 (MK-7).

In nature, vitamin K occurs in two main forms namely, phylloquinone (vitamin K1) and various menaquinones (vitamin K2) (Mahdinia et al., 2017). Vitamin K2 is a fat-soluble vitamin that has an ability to cross the blood brain barrier (BBB) due to its lipophilic nature (Talaei Firozjaei et al., 2018). All the existing forms of vitamin K2 consist of a common moiety in their chemical structure which is a 2-methyl-1,4-naphthoquinone ring. This core chemical structure of vitamin K2 is called menadione (Figure 1). Each form differs from each other with respect to their degree of saturation and length of side chains (Iwamoto, 2014). Menaquinone (also referred to as MK-n) differs in the length of its side chain varying from 4 to 15 isoprene units; n denotes the number of isoprene units present in the structure (Beulens et al., 2013). Figure 2 depicts the chemical structures of members of the vitamin K2 family. The differences in side chains of different forms give each of them a unique potency and absorption efficiency (Booth and Rajabi, 2008). Menaquinones with absence of methyl group on naphthoquinone moiety at position 3 are called dimethyl-menaquinones and serve as precursors to menaquinones (Bentley and Meganathan, 1982).

### Sources of K2-7

Main sources of phylloquinone include plant-based foods like leafy vegetables, fruits, *etc*. whereas sources of menaquinones include animal-based foods like meat, fishes, dairy products, *etc*. Short chain menaquinones like MK-4 (having four isoprene units) are found majorly in animal-based food products. Menaquinones having long chains such as MK-7 to MK-13 are primarily synthesized by bacteria (Shea and Booth, 2016) that include species of aerobic, anaerobic, facultative, as well as obligate anaerobic bacterial species that include *Bacillus*, *Corynebacterium*, *Escherichia*, *Lactococcus* and *Vibrio*. Some of these bacterial species are present as microflora in the gut (Bentley and Meganathan, 1982), (Conly and Stein, 1992), (Walther et al., 2013). MK-7 or K2-7 is found to be present in very high concentration in a Japanese traditional food called natto. Natto is made by fermenting soybean using *Bacillus subtilis*. Another source of menaquinones is the microflora of intestine. Although intestinal synthesis has been shown to produce significant quantities of menaquinones, absorption from this source is inefficient to fulfill the required quantities. Menaquinone is analogous to ubiquinone in function as ubiquinone also contains isoprenoid side chains of varying length, and therefore is used as an electron carrier in electron transport chain by bacteria. Depending on the dietary intake, the presence of vitamin K2-7 is also detected in human milk samples (Marles et al., 2017).

### Dietary Intake of K2-7

As previously mentioned, menaquinones are originated from microbial species, and therefore, their sources majorly include dairy products and foods that are fermented exclusively by bacteria, like in natto (whole soyabeans that are fermented) (Beulens et al., 2013). The levels of K2-7 are highest in natto; moderate in chicken, sauerkraut, beef and a variety of cheeses; and low in pork, salmon, *etc* (Halder et al., 2019). Dietary intake of a population differs regionally as there is a difference of menaquinone content in food with respect to the form present in the food and the amount consumed. Mostly, availability of K2-7 from diet is low globally except in few geographic regions such as Japan and western Europe. In Japanese population, K2-7 intake is mainly due to the consumption of natto; dairy intake of western Europe population is high containing K2-7 in the fermented natural cheese, further, other forms of vitamin K2 are present in dairy products. Among all dairy products, the most important source of menaquinones is cheese. As different bacteria are used for cheese production, the content of menaquinone shows a wide variability in every cheese type (Fox et al., 2004).

### Production of K2-7

Production of menaquinones is observed in bacterial cells and in animals as well. In humans, anaerobes like *Escherichia coli* present in large intestine synthesizes long chain derivatives of menaquinones (MK-7 to MK-11). Intestinally synthesized menaquinones are used as electron transporters in anaerobic respiration. Menaquinones are also produced commercially by the process of fermentation (solid state fermentation (SSF) or liquid state fermentation (LSF)) or by chemical synthesis. In SSF, the water content ranges up to 12–80%, whereas the water content is very high i.e., up to 90–95% in LSF (Mitchell et al., 2000). SSF is used for the production of secondary metabolites; the raw material used here is soy protein and corn grits. Various factors affect the production of vitamin K2-7 in SSF such as the microbial strain used, raw material or substrate along with its pre-treatment, temperature and time for which the fermentation is run. Pre-treatment of substrate is an important factor as it can increase the yield of the production. Pre-treatment using α-amylase enzyme at the first step in the fermentation process results in rise of sugar monomers and likely increases yield as well (Mahanama et al., 2011). Another important factor in the fermentation process is the selection of the bioreactor. Tray bioreactors, static deep bed bioreactors and dynamic mode fermenters that include rotating drums are some of the bioreactors used for SSF (Mahdinia et al., 2017).

Currently, liquid fermented products enjoy the favor of commercial markets. Several modifications have been researched in LSF for the fermentation media or the microbial strain or mechanical design to improve the vitamin K2-7 yield and reduce the fermentation time. Mainly *Bacillus* species including *B. subtilis* and *B. licheniformis* are studied for LSF (Mahdinia et al., 2017). Morishita *et al.* (Morishita et al., 1999) used lactic acid bacteria for the production of all vitamin K2 forms. *Leuconostoc lactis* and *Lactococcus* strains including *L. lactis* ssp. *lactis* and *L. lactis* ssp. *cremoris* produced quinones when grown on medium containing soy milk. Therefore, these strains are used as starter cultures in dairy (Morishita et al., 1999).

Ranmadugala *et al.* studied the *Bacillus subtilis* fermentation for high production of K2-7 using biocompatible organic solvents, n-hexane and n-butanol mixture. A ∼1.7-fold increase of total K2-7 was observed when compared to the control. Further, effect of 3-aminopropyltriethoxysilane-coated ferric oxide nanoparticles was studied on K2-7 production using *Bacillus subtilis* (ATCC 6633). The production as well as yield of K2-7 showed a significant increase when the strain was treated with 3-aminopropyltriethoxysilane-coated ferric oxide nanoparticles. An increase in yield by 2-fold was obtained compared to the fermentation medium containing untreated bacterial strain (Ranmadugala et al., 2018). Degree of saturation and number of isoprene units vary with the organism that synthesizes the compound (Beulens et al., 2013).

Synthesis of vitamin K2-7 results in the formation of two isomers, cis-isomer and trans-isomer. Among both the isomers, only trans-isomer has a role in biological activities. The precursor for synthesizing vitamin K2-7 is menadione, but it has harmful effects on humans. To overcome this drawback, Li *et al.* used *Pichia pastoris* strain and biologically transformed toxic menadione into vitamin K2-3 (MK-3) as an alternate precursor for the fermentation process of vitamin K2-7 (Li Z. et al., 2017). *Bacillus subtilis natto* is proven to be a promising microorganism for producing vitamin K2-7 on an industrial scale, as vitamin K2-7 produced is secreted extracellularly as well as intracellularly. Extracellularly secreted vitamin K2-7 is bound with vitamin K2-binding factor, a protein complex, which is soluble in the fermentation broth. Biosynthesis pathway initiates with shikimic acid and ends at vitamin K2-7 involving seven intermediates. Fermentation in static culture results in formation of biofilm and growth in the form of pellicle (Mahdinia et al., 2019).

Mahdinia *et al*. studied the key growth factors required for improving the production of vitamin K2-7 in biofilm reactors using *B. subtilis natto*. The growth factors included optimum pH, temperature and agitation. The medium used for the production was glycerol-based as it is relatively cheaper compared to other carbon sources like sucrose, glucose and mannose and can also improve the production of vitamin K2-7. The optimum growth parameters reported were pH: 6.58, temperature: 35°C and agitation: 200 rotations per minute (rpm). An increase of 58% in the concentration of vitamin K2-7 was observed when growth parameters were optimized (Mahdinia et al., 2018).

**FIGURE 3:**
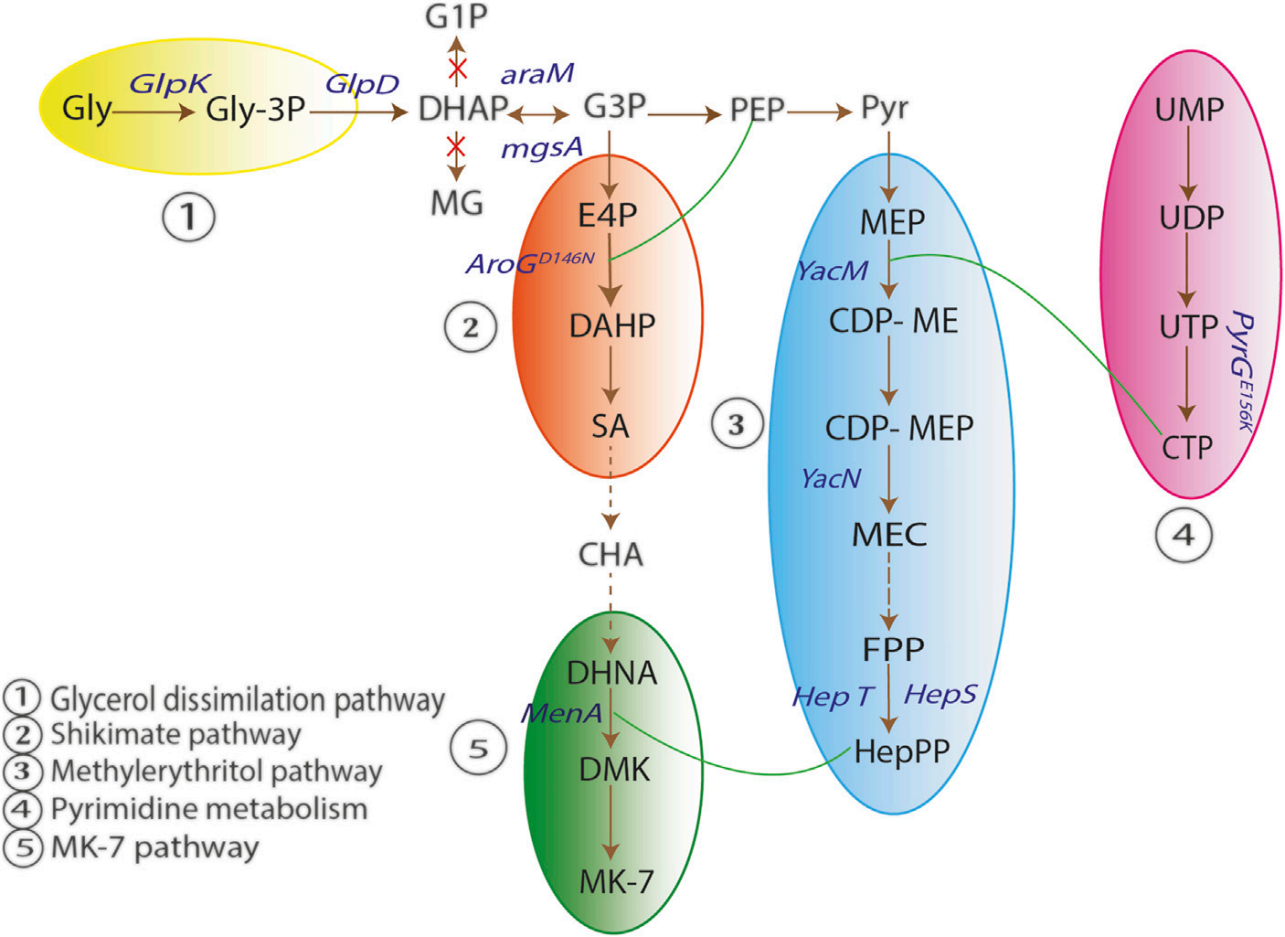
Schematic representation of vitamin K2-7 synthesis. In glycerol dissimilation pathway, overexpression of enzymes glycerol kinase (GlpK) and glycerol-3-phosphate dehydrogenase (GlpD) leads to conversion of glycerol (Gly) to dihydroxyacetone phosphate (DHAP). Conversion of DHAP to methylglyoxal (MG) and glyceraldehyde-1-phosphate (G1P) is prevented by deleting mgsA and araM. DHAP is interconverted to glyceraldehyde-3-phosphate (G3P) which is converted to pyruvate (Pyr). Pyr enters the methylerythritol pathway (MEP) in which the end product is heptaprenyl pyrophosphate (HepPP). Synthesis of HepPP is enhanced by overexpression of enzyme heptaprenyl diphosphate synthase (HepS). HepPP enters MK-7 pathway to form MK-7 (vitamin K2-7). AroG^D146N^ and PyrG^E156K^ are overexpressed in shikimate (SA) pathway and pyrimidine metabolism pathway respectively to lead the components of both the pathways towards MK-7 pathway and form MK-7 as the end product (Adapted from (Yang et al., 2020)).

Yang *et al.* constructed a metabolically engineered *B. subtilis* strain for enhancing the production of MK-7. In *B. subtilis*, synthesis of vitamin K2-7 is divided into five pathways, *viz*., glycerol dissimilation/dissociation pathway, shikimic acid (SA) pathway, methylerythritol phosphate (MEP) pathway, pyrimidine metabolism pathway and vitamin K2-7 (MK-7) pathway. In glycerol dissimilation pathway, overexpression of enzymes like glycerol kinase (GlpK) and glycerol-3-phosphate dehydrogenase (GlpD) led to conversion of glycerol to dihydroxyacetone phosphate (DHAP). Further, conversion of DHAP into methylglyoxal (MG) and glyceraldehyde-1-phosphate (G1P) was prevented by deleting *mgsA* and *araM*. DHAP is interconverted to glyceraldehyde-3-phosphate (G3P) which further converts into pyruvate and enters MEP pathway wherein the end product is heptaprenyl pyrophosphate (HepPP). Synthesis of HepPP was enhanced by overexpressing enzyme heptaprenyl diphosphate synthase (HepS). HepPP then entered MK-7 pathway to form vitamin K2-7. In addition, *AroG* ^*D146N*^ and *PyrG* ^*E156K*^ were overexpressed in SA pathway and pyrimidine metabolism pathway respectively to lead the components of both the pathways towards MK-7 pathway and ultimately form MK-7 as the end product in high concentrations as shown in Figure 3 (Yang et al., 2020).

Gao *et al*. reported the use of genetically and metabolically engineered *Escherichia coli* for production of vitamin K2-7. Under aerobic conditions, ubiquinone-8 (Q-8) is produced by *E. coli*, and, under anaerobic conditions, it produces vitamin K2-8 instead of vitamin K2-7, because of the presence of octaprenyl diphosphate synthase which is encoded by *IspB*. For synthesizing one molecule of vitamin K2-7, seven molecules of isopentenyl diphosphate (IPP), the building blocks of isoprenoid units, are required. In mevalonic acid pathway, acetyl-CoA is converted to IPP which is further converted into vitamin K2-7 *via* heptaprenyl pyrophosphate. This study was carried out to produce engineered *E. coli* by introducing heptaprenyl pyrophosphatase synthetase (HepPPS) enzyme derived from *B. subtilis*. Further, production of vitamin K2-7 was enhanced by overexpressing HepPPS and optimizing the expression of enzymes in mevalonic acid pathway in order to eliminate synthesis of metabolites other than those involved in K2-7 pathway (Gao et al., 2020). In another study Gao *et al.* investigated an alternative procedure for highly efficient production of vitamin K2-7 by using metabolically engineered *E. coli* under aerobic conditions (Gao et al., 2021).

We have enlisted the synthetic pathways involved in production of vitamin K2-7 in Table 1 along with their advantages and disadvantages.

**TABLE 1:** Synthetic pathways in production of vitamin K2-7.

Synthetic pathway	Advantages	Disadvantages	References
**A. Genetic and metabolic engineering**			
*1. E. coli (*pLB1s-ESK1)	High yield of MK-7; has a short production cycle	Requires optimization	Gao et al. (2020)
*2. B. subtilis*	Best MK-7-producing recombinant microbe	High cost of *B. subtilis* fermentation; has a long production cycle	(Ma et al., 2019; Gao et al., 2020)
**B. Fermentation**			
1. Soy protein granules and nixtamalized corn grits	Higher yield of MK-7 and low manufacturing costs	—	Mahanama et al. (2011)
*2. Lactococcus lactis ssp. cremoris, Lactococcus lactis ssp. lactis, Leuconostoc lactis*	Higher yield of MK-7	—	Morishita et al. (1999)
*3. B. subtilis natto*	Improved production of MK-7	Formation of pellicles which can be problematic and undesirable during fermentation	(Berenjian et al., 2013)

## Vitamin K2-7 and Associated Diseases

Vitamin K2-7 plays an important role in various biological functions along with vitamin K-dependent proteins (VKDPs). K2-7 is a cofactor of enzyme γ-carboxylase which drives the conversion of inactive VKDPs (such as osteocalcin and matrix Gla protein) to their active forms. Vitamin K2 helps in carboxylation of glutamate (Glu) residues present on VKDPs to γ-carboxyglutamate (Gla) which results in their activation. In the absence of vitamin K2, the optimal functioning of these proteins is hindered which leads to pathological complications that include metabolic conditions like diabetes and chronic degenerative conditions (cardiovascular disease, osteoporosis, *etc*) (Vaidya et al., 2022b). Genes modulated by vitamin K2-7 in various diseases are summarized in Table 2.

**TABLE 2:** Genes modulated by vitamin K2-7 in various diseases.

Conditions	Genes	Expression	References
Osteoporosis	*biglycan*	Downregulated	Katsuyama et al. (2007)
	*butyrophilin*	Downregulated	Katsuyama et al. (2007)
	*tenascin C*	Upregulated	Katsuyama et al. (2007)
	*BMP2*	Upregulated	Katsuyama et al. (2007)
Vascular calcification	*BMP2*	Downregulated	Boström et al. (1993)
Cancer	*cyclin D1*	Downregulated	Xia et al. (2012)
Diabetes	*TNFα*	Downregulated	Li et al. (2018)
	*IL-1β*	Downregulated	Li et al. (2018)
	*IL-6*	Downregulated	Li et al. (2018)
Peripheral neuropathy	*TNFα*	Downregulated	Pan et al. (2016)
	*IL-1β*	Downregulated	Pan et al. (2016)
Alzheimer’s disease	*TNFα*	Downregulated	Saputra et al. (2019)
	*IL-1β*	Downregulated	Saputra et al. (2019)
	*IL-6*	Downregulated	Saputra et al. (2019)

### Vitamin K2-7 and Osteoporosis

Osteoporosis is the most common bone disorder found in the older population (Mandatori et al., 2021). An imbalance between bone formation and bone resorption causes this metabolic disorder leading to depletion of bone mass, deterioration of skeletal structure and an elevated risk of bone fractures (Hendrickx et al., 2015). Bone homeostasis is maintained by osteocytes, osteoblasts and osteoclasts. Bone formation is promoted by osteoblasts and bone resorption is stimulated by osteoclasts. Decreased formation and increased resorption of bone results in bone loss with aging. Osteoporosis is caused by bone loss along with various pathophysiologic states. K2-7 aids in stimulation of osteoblastic formation of bone and suppression of osteoclastic resorption of bone. In osteoblastic cells, K2-7 helps in protein synthesis of osteocalcin and various other proteins. Interestingly, the ratio of circulating K2-7 serum levels between eastern Japanese women and British women is 15:1 which inversely mirrors the fracture rate of 1 in Japanese women to that of 15 in British women (Kaneki et al., 2001). This implies a strong beneficial effect of K2-7 circulating levels in reducing the risk of fractures globally.

**FIGURE 4:**
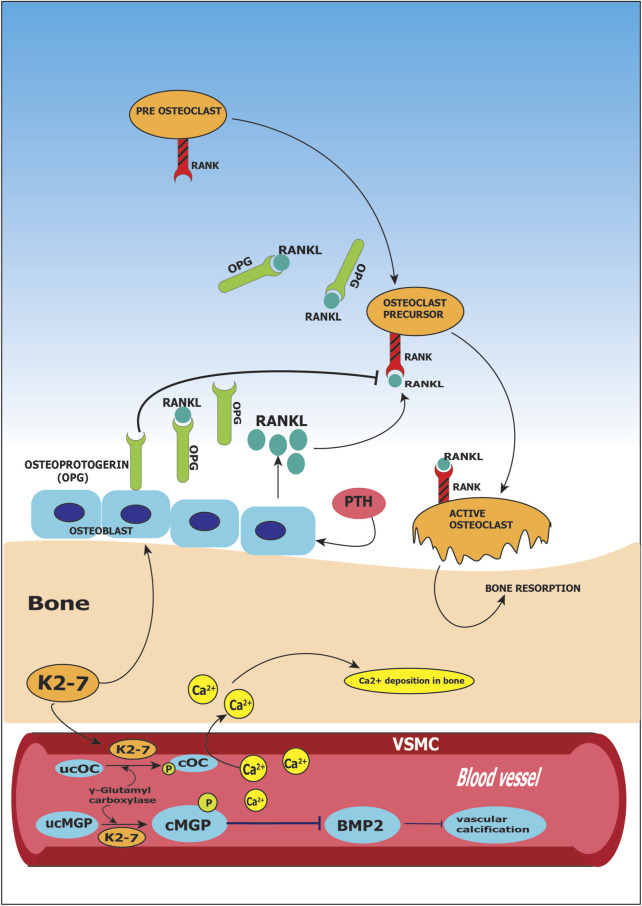
Role of vitamin K2-7 in osteoporosis and vascular calcification. Vitamin K2-7 facilitates carboxylation of glutamate (Glu) residue present on matrix Gla protein (MGP) leading to formation of carboxylated MGP (cMGP) and aids in phosphorylation of MGP leading to its activation; activated MGP inhibits bone morphogenetic protein 2 (BMP2) resulting in prevention of vascular calcification. K2-7 also is a cofactor in conversion of undercarboxylated osteocalcin (ucOC) to carboxylated osteocalcin (cOC); cOC has an affinity for calcium ions and facilitates the transport of calcium to bone for bone formation. Receptor activator for nuclear factor kappa ligand (RANKL) binds to RANK receptor and activates osteoclasts which results in bone resorption; vitamin K2-7 augments the expression of osteoprotegerin (OPG) which is a decoy receptor for RANKL and abrogates RANK-RANKL binding thus inhibiting bone resorption.

Cellular functions in osteoclastic and osteoblastic cells are performed by various proteins, whose expression is regulated by K2-7 (Yamaguchi, 2014). Osteocalcin produced by osteoblasts binds to calcium present in blood circulation and leads it to the bone matrix. Bone mineralization is influenced by osteocalcin as it has high affinity towards hydroxyapatite, a mineral component of bone; this results in stronger skeleton and less susceptibility to fracture (Hoang et al., 2003). The newly synthesized osteocalcin is inactive and it requires vitamin K2-7 for converting itself into active form by carboxylation, and later bind to calcium (Hauschka et al., 1989). Vitamin K2-7 is a cofactor of enzyme γ-carboxylase, that converts glutamic acid (Glu) residues present in the molecule of osteocalcin (OC) to γ-carboxyglutamate (Gla) and is, therefore, necessary for the γ-carboxylation of OC. Thus, vitamin K2-7 is thought to be involved in bone mineralization as an essential element for the γ-carboxylation of OC (Iwamoto et al., 2006). K2-7 by upregulating osteoprotegerin, a decoy receptor for RANKL, prevents bone resorption. RANKL otherwise binds to receptor activator of NF-κB (RANK) and activates nuclear factor kappa beta (NF-κB). Activation of NF-κB is essential for osteoclasts proliferation which results in osteoporosis through bone resorption. Thus, RANK receptor is sequestered by K2-7 preventing steps leading to osteoporosis shown in Figure 4 (Badmaev et al., 2011).

Wu *et al.* performed a comparative study of vitamin K2-7 effect, isolated from *cheonggukjang*, with vitamin K2-4 and vitamin K1. The effect of all three vitamins was determined on mineralization and cell differentiation of the pre-osteoblastic cell line MC3T3-E1 obtained from neonatal mouse calvariae. Vitamin K2-7, vitamin K2-4 and vitamin K1 significantly enhanced activity of alkaline phosphatase (ALP) that resulted in bone formation; increased proliferation of osteoblastic cell line MC3T3-E1 was also reported. It was observed that vitamin K2-4 and K2-7 had comparatively more dynamic effect than vitamin K1; this suggested that vitamin K2 (K2-4 and K2-7) and vitamin K1 probably have different mechanisms for stimulation of mineralization of osteoblasts. Furthermore, the mRNA expression ratio of osteoprotegerin and RANKL was also upregulated after treatment with 10 µM of vitamin K2 (K2-4 and K2-7) for 24 h. This indicates that vitamin K has a potential role in suppression of osteoclast formation by upregulating mRNA expression ratio of osteoprotegerin and RANKL (Wu et al., 2019).

Katsuyama *et al.* (Katsuyama et al., 2007) using MC3T3E1 osteoblastic cells obtained from calvaria of newborn mouse studied the differential expression of genes after treatment with vitamin K2-7. Both control and vitamin K2-7-treated MC3T3E1 cells were analyzed. Downregulation of *biglycan* and *butyrophilin* genes and upregulation of *tenascin C* and *BMP2* were observed after 24 h of treatment with K2-7. Tenascin C is an important protein involved in bone remodeling as it promotes differentiation of osteoblastic cells. BMP2 signaling is responsible for the production of osteoblast-specific proteins such as osteocalcin. Indeed, MK-7 induces the production of osteoprotegerin, osteocalcin receptor activator of NFκB (RANK), and its ligand (RANKL) in osteoblastic MC3T3E1 cells, thus indicating that vitamin K2 may play an important role in bone homeostasis. Biglycan is a member of the class I family of small leucine rich proteoglycans (SLRPs). Studies have established the role of biglycan in osteoblast differentiation and matrix mineralization through the BMP4 signaling pathway (Nastase et al., 2012). *In vitro* studies have shown that biglycan interacts with other BMPs, such as BMP2 and 6. Biglycan is able to directly bind BMP2 and its receptor, ALK6 (also known as BMP-RIB), to stimulate BMP2- dependent osteoblast differentiation (Moreno et al., 2005). Butyrophilin is associated in regulating B7 protein (Malinowska et al., 2017) which is involved in osteoblast differentiation (Suh et al., 2004, 3).). It was observed that vitamin K2-7 increased tenascin C levels through BMP2 pathway which has a role in autocrine signaling. Tenascin C levels increased by ∼1.5 fold when treated with 10^–5^ M vitamin K2-7 and ∼3 fold when treated with 10^–6^ M of vitamin K2-7. In addition, increase in phosphorylated Smad1 level was reported in cells treated with 10^–6^ M vitamin K2-7. As nuclear binding receptor for vitamin K2-7 is present on MC3T3E1 cells, it is possible that, through production of BMP2, K2-7 has an indirect effect on the BMP2-Smad1 pathway.

Gigante *et al.* (Gigante et al., 2015) studied the effect of vitamin K2-7 (with and without vitamin D3) on differentiation of human mesenchymal stem cells (hMSCs) obtained from bone marrow. Vitamin K2-7 enhances the process of gene induction of osteocalcin that is initially influenced by vitamin D3. It was observed that vitamin K2-7 has an effect on genes involved in cell growth and cell differentiation which included growth differentiation factor-10 (*GDF10*) and insulin-like growth factor 1 (*IGF1*). In addition, co-supplementation of vitamin K2-7 influenced vascular endothelial growth factors (VEGFA) induction along with its receptor fms-related tyrosine kinase 1 (FLT1); FLT1 plays a role in both angiogenic and osteogenic processes. Co-supplementation of vitamins aided in bone-healing process by modulating the expression of genes involved in both mineralization and angiogenesis. Considering genes involved in bone formation and mineralization, vitamin K2-7 enhanced vitamin D3 gene induction of osteocalcin. Hence, co-supplementation strategy has the potential to help in better development of bone and reduce bone-related disorders.

### Vitamin K2-7 and Vascular Calcification

Vascular calcification is characterized by mineral depositions on the walls in the vascular system; the depositions present are of calcium phosphate complexes in the form of hydroxyapatite (El Asmar et al., 2014). Based on recent clinical and pre-clinical studies, Abdullah et al. highlighted the potential cellular and physiological roles of vitamin K in cardiovascular diseases (CVD), and highlighted the association between CVD prevention and vitamin K supplementation (Al-Suhaimi and Al-Jafary, 2020). Matrix Gla protein (MGP) plays a key role in inhibition of vascular calcification; it has the potential to inhibit as well as to reverse the process of calcification. MGP undergoes post-translational modifications like phosphorylation and carboxylation which result in the activation of MGP from its inactive state. Serine residues present on MGP are phosphorylated by casein kinase, this results in secretion of MGP in the extracellular matrix (Roumeliotis et al., 2019). In addition, activation of MGP takes place when glutamate residues present on MGP undergo carboxylation by γ-carboxylase. Vitamin K2-7 is the cofactor of γ-carboxylase, hence, plays an important role in activation of MGP. Negatively charged MGP has high affinity towards free calcium present in the blood vessels. It binds directly to the circulating calcium and hydroxyapatite crystals that are accumulated in the walls of the vessels forming inactive complexes (Roumeliotis et al., 2020). Negatively charged MGP has high affinity towards free calcium present in the blood vessels. It binds directly to the circulating calcium and hydroxyapatite crystals that are accumulated in the walls of the vessels forming inactive complexes (Roumeliotis et al., 2020). MGP initiates autophagic clearance by attracting macrophages and phagocytes (Shanahan, 2005). In addition, MGP removes free circulating calcium and leads it to the bone. Furthermore, MGP inhibits vascular calcification through downregulation of bone morphogenetic protein-2 (BMP-2) which promotes vascular calcification. Transformation of VSMCs to an osteoblastic phenotype is triggered by BMP-2 and is found within the walls of calcified arteries (Boström et al., 1993). MGP is in inactive state and requires vitamin K2-7 for γ-carboxylation of its glutamic acid (Glu) into γ-carboxyglutamate (Gla). When Glu is transformed into Gla, molecular changes occur in the structure of MGP which in turn activates it. Further, to become biologically active, MGP undergoes phosphorylation of serine residues. Phosphorylation is dependent on vitamin K and is considered as the most critical step in activation of MGP. Therefore, to gain the ability to bind to hydroxyapatite, calcium and BMP-2 MGP is required to be carboxylated as well as phosphorylated (Figure 4) (Wallin et al., 2000). Furthermore, vascular calcification is also associated with activation of growth arrest-specific gene 6 (Gas6) activated by vitamin K2. Gas6 undergoes γ-carboxylation by vitamin K2 to trigger anti-apoptotic activity of Bcl-2. Gas6 also inhibits caspase 3, a pro-apoptotic protein, thus preventing the apoptosis induced by calcification and starvation of fibroblasts (Villa et al., 2017). Gas6 and other growth factors act as growth promoters in VMSCs.

Vascular calcification is a significant predictor of cardiovascular disease. As mentioned earlier, MGP plays a crucial role in inhibition of vascular calcification. Through γ-carboxylation, vitamin K2 may help reduce vascular calcification and thereby reduce the risk of cardiovascular disease (Beulens et al., 2013). Patients suffering from chronic kidney disease often build up complications like vascular calcification and bone disorders that are caused by mineral disturbances (Schlieper et al., 2016). Thus, phosphate retention occurs as the function of kidney becomes less efficient. For the transformation of VMSCs into osteoblast-like cells, phosphate plays a role as key signaling mediator. Arterial wall mineralization is mediated by bone matrix proteins that are produced by osteoblast-like cells (Leopold, 2015). Vitamin K2 carboxylates MGP to prevent vascular calcification and in turn abrogates complications in chronic kidney disease.

### Vitamin K2-7 and Cancer

Several therapeutics are available for patients suffering from cancer, yet prognosis for the long term is inadequate for different types of cancer. Vitamin K2 exerts anti-cancer effects on various cell lines such as leukemia, hepatocellular carcinoma (HCC), lung cancer, ovarian cancer, pancreatic cancer and colorectal cancer (Xv et al., 2018). Cancerous cell growth is suppressed by vitamin K2 *via* apoptosis, autophagy and cell-cycle arrest.

Vitamin K2 has the ability to inhibit proliferation of cancer cells through induction of cell-cycle arrest, here the activity of NF-κB is inhibited which plays an important role. NF-κB is a nuclear transcription factor and associates itself with cell growth by regulation of the *cyclin D1* gene (Xia et al., 2012). During cell cycle, Cyclin D1 contributes to the G_1_-S transformation by binding to CDK4 or CDK6 (Masaki et al., 2003). The expression of cyclin D1 is downregulated by vitamin K2 through preventing NF-κB from binding to cyclin D1 promoter (Xia et al., 2012). Another mechanism by which vitamin K2 shows its anticancer effect is by inducing apoptosis of cancer cells. Mitochondrial apoptosis is induced by vitamin K2 *via* mitogen-activated protein kinase (MAPK) pathways (Tsujioka et al., 2006; Kanamori et al., 2007; Sibayama-Imazu et al., 2008; Showalter et al., 2010). Extracellular signal-related kinases (ERKs) stimulate cell proliferation and inhibit cell-death signals in response to mitogenic signals or growth factors whereas, c-Jun N-terminal kinase (JNK) pathway and p38 MAPK pathway are implicated in apoptosis and inflammation. Activation of p38 is carried out by vitamin K2 by phosphorylating p38 (Olson and Hallahan, 2004; Tsujioka et al., 2006). Vitamin K2 can inhibit cancer cell growth by inducing autophagy. Vitamin K2 has the ability to simultaneously cause autophagy and apoptosis in leukemic cells; when high expressions of Bcl-2 are observed, autophagy is comparatively more dominant, this restrains apoptosis. Therefore, autophagy can be an alternative for inducing apoptosis instead of distinct form of cell death (Yokoyama et al., 2008).

In the cell cycle, proto-oncogenic protein Cyclin D1 regulates the G_1_ to S phase transition by phosphorylation of pRb and binding to Cdk4 or Cdk6 (Hall and Peters, 1996). Synthesis of DNA and contact-independent growth in several human cancers including hepatocellular carcinoma (HCC) can be enhanced when cyclin D1 is overexpressed (Zhang et al., 1993; Hall and Peters, 1996; Masaki et al., 2003). Expression of *Cyclin D1* gene is regulated by NF-κB along with several other factors (Guttridge et al., 1999; Joyce et al., 2001). Ozaki *et al.* (Ozaki et al., 2007) investigated the effect of vitamin K2 on growth inhibition of HCC cells (Huh7, HepG2 and Hep3B). Expressions of growth-related genes that included cyclin D1 and cyclin-dependent kinase inhibitors were evaluated at the protein and mRNA levels after HCC cells were treated with different concentrations of vitamin K2 ranging from 10^–4^ to 10^–7^ M for 48 h. G_1_ phase arrest was observed in HCC cells treated with vitamin K2. Cyclin D1 protein and mRNA expression was downregulated by vitamin K2 treatment. Moreover, the expressions of Cdk inhibitors (p21 and p27) were elevated in HepG2 cells. This indicated that during vitamin K2-induced growth inhibition in HCC cells, the expressions of cyclin D1 and Cdk inhibitors is regulated. Activity of cyclin D1 promoter was examined to further study the effects of vitamin K2. Vitamin K2 inhibited the cyclin D1 promoter activity significantly in all the three cell lines in a dose-dependent manner. Inhibition of activity of cyclin D1 promoter by vitamin K2 is dependent on NF-κB. Vitamin K2 also contributed to the inhibition of both basal and induced NF-κB binding activities by inhibiting the activity of inhibitory κB kinase (IKKα). Hence, vitamin K2 inhibited HCC cells growth by suppressing expression of cyclin D1 *via* IKK/NF-κB pathway and, therefore, can prove to be useful for treating HCC.

The post-translational carboxylation of the prothrombin precursor is dependent on vitamin K-dependent γ-glutamyl carboxylase. HCC cells are unable to carboxylate all Glu residues due to carboxylase deficiency (Uehara et al., 1999). Prothrombin Induced by Vitamin K absence-II (PIVKA-II), also known as des-gamma-carboxy prothrombin (DCP), is an abnormal prothrombin precursor produced in HCC. It is a potential autologous growth stimulator for HCC proliferation and has insufficient coagulant activity. PIVKA-II is considered as a potential serum biomarker for HCC with better diagnostic performance and higher specificity in early detection of HCC (Feng et al., 2021). PIVKA-II in combination with serum α-fetoprotein (AFP) enhanced the accuracy of surveillance of HCC in high-risk populations. Additionally, PIVKA-II acts as a marker for the assessment of response to treatment in HCC (Yang et al., 2021). Hitomi et al. investigated the antitumor effects of vitamin K2 on athymic BALB/c-nu/nu mice. Vitamin K2 (400 µM) was administered orally for 53 days from the day after inoculation of PLC/PRF/5 human HCC cells. It was observed that there was significant decrease in tumor size (five fold). In addition, there was significant reduction in the protein expression of cyclin D1 and Cdk4 in HCC suggesting G1 arrest of the cell cycle. However, Cdk inhibitor p16^INK4a^ was not affected by vitamin K2 (Hitomi et al., 2005; Al-Suhaimi, 2014). Furthermore, Lu et al. studied the inhibition of HCC cell proliferation using vitamin K2 in HepG2 cells. 17β-hydroxysteroid dehydrogenase 4 (HSD17β4) is a protein that promotes cell proliferation. When HSD17β4 is overexpressed, it promotes the cell proliferation of HCC. Vitamin K2 binds directly to the protein, without affecting its gene expression, and inhibits MEK/ERK and Akt signaling pathways which further result in inhibition of HCC cell proliferation (Lu et al., 2021).

### Vitamin K2-7 and Diabetes

Diabetes mellitus type 2 (T2DM) which is associated with macro- and micro-vascular complications is a major public health problem around the world. We have recently delineated the noncoding RNA interactome including microRNAs (miRNAs) and long noncoding RNAs (lncRNAs) in the pathogenesis of diabetes (Pandey et al., 2022). In T2DM, insulin sensitivity is improved by vitamin K2 through involvement of osteocalcin (vitamin K-dependent protein), anti-inflammatory properties, and lipid-lowering effects (Zhang et al., 2017; Li et al., 2018). Vitamin K2 improves sensitivity to insulin in diabetic patients *via* metabolism of osteocalcin which has a role in increasing adiponectin expression. In addition, administration of vitamin K2 results in alteration of NF-κB and GSK-3β expression involved in chronic inflammation and adiponectin regulation respectively (Otani et al., 2015; Zhang et al., 2017). Undercarboxylated osteocalcin (ucOC) is an active hormone that affects glucose metabolism. It is an important biomarker for vitamin K status and is associated with diabetes (Lin et al., 2018). Supplementation of vitamin K2 increased carboxylation of osteocalcin (Berkner, 2005), and it was postulated that osteocalcin influenced insulin sensitivity *via* adiponectin regulation. Osteocalcin signaling pathway regulated glucose metabolism which led to an increase in insulin1 (Ins1) and insulin2 (Ins2) gene expression (Hussein et al., 2018; Al-Suhaimi and Al-Jafary, 2020). In the liver, phosphorylation of peroxisome proliferator-activated receptors (PPAR-α), p38 MAP kinase and 5′AMP-activated protein kinase (AMPK) is increased as adiponectin receptor 2 mediates the effect of adiponectin. In addition, the effect of globular adiponectin is controlled by adiponectin receptor 1 resulting in increased phosphorylation of PPAR-α, p38 MAPK and AMPK. It was observed that insulin sensitivity was also augmented with increase in oxidation of fatty acids and glucose uptake (Weyer et al., 2001; Yamauchi et al., 2003; Lihn et al., 2005). Response to inflammation has a role in etiology as well as pathogenesis of T2DM. IL-6, a proinflammatory mediator, plays a role in activation of suppressor of cytokine signaling (SOCS) protein, this in turn blocks the activation of insulin transcription factor. Signal transducer and activator of transcription 5B (STAT5B) protein binds to insulin receptor and activates insulin transcription factor. SOCS competes with STAT5B that blocks the activation of insulin transcription factor (Emanuelli et al., 2000). Vitamin K2 inhibits inflammatory responses *via* NF-κB signaling pathway inactivation, which plays a key role in suppressing the expression of IL-6, IL-1β, TNF-α. Thus, by suppressing inflammatory responses, vitamin K2 improves insulin resistance (Li et al., 2018).

### Vitamin K2-7 and Peripheral Neuropathy

Peripheral neuropathy is a disorder characterized by numbness, slow nerve conduction, pain and tingling sensations in the limbs (Head, 2006). One of the causes of peripheral neuropathy is demyelination of peripheral nerve fibres. Demyelination results in deterioration of the structural and molecular features of the nerve fibres, which leads to peripheral neuropathy (Wei et al., 2019). We have recently reviewed the intrinsic need for vitamin K2-7 supplementation in peripheral neuropathy (Vaidya et al., 2022a). We have also recently elucidated the miRNA regulatory interactome in neuropathic pain (Gada et al., 2022). Vitamin K2 facilitates synthesis and repair of the myelin sheath in the peripheral nervous system. In addition, vitamin K2 facilitates the activation of Gas6 by carboxylating the Gla residues of Gas6, which is structurally related to anticoagulation factor protein S. Gas6 and protein S bind to form a complex and activate the receptor tyrosine kinase of TAM (Tyro3, Axl, and Mer) family which leads to increased myelin production and repair after myelin injury (Mehta, 2017). Proinflammatory cytokines like TNFα and IL-1β are associated with peripheral neuropathy development through neuroinflammatory mechanisms (Li QY. et al., 2017). Vitamin K2 is able to inhibit gene expression of TNFα and IL-1β in human monocyte-derived macrophages in a dose-dependent manner (Pan et al., 2016). In bones, primary activation of the RANKL/RANK (receptor activator for nuclear factor kappa B) system activates osteoclasts, which triggers the damage of bones and subsequently cause damage to the peripheral sensory nerves around bones due to bone fracture. Neuropathic pain develops when there is a damage in peripheral nerves (Zajączkowska et al., 2019). RANKL/RANK system activates NF-κB, a regulator of inflammation, leading to osteoclasts activation. Vitamin K2-7 prevents activation of the RANKL/RANK system by upregulating osteoprotegerin, a decoy receptor for RANKL. Thus, by preventing the binding of RANKL to the RANK receptor, vitamin K2 abrogates activation of NF-κB and hence activation of osteoclasts (Badmaev et al., 2011). Considering these properties of vitamin K2-7, it may likely serve as an effective intervention in the management of neuropathic pain.

### Vitamin K2-7 and Alzheimer’s Disease

According to Alzheimer’s Foundation of America, “Alzheimer’s disease is a progressive brain disorder that impacts memory, thinking and language skills, and the ability to carry out the simplest tasks.” (Alzheimer’s Foundation of America). The levels of vitamin K2 in serum level were reduced in patients suffering from AD (Huy et al., 2013). Vitamin K2 has the potential to slow down the progression of AD and contribute to its prevention.

Hadipour *et al.* (Hadipour et al., 2020) studied the effects of vitamin K2 at concentrations ranging from 5 to 200 μM in rat pheochromocytoma PC-12 cells to provide protection against toxicity caused by hydrogen peroxide and β-amyloid. Vitamin K2 reduced the cytotoxicity caused by hydrogen peroxide and β-amyloid. In Alzheimer’s disease, β-amyloid led to neuronal death by direct toxicity and by promotion of apoptosis; whereas vitamin K2 prevented neuronal death resulting from β-amyloid in PC-12 cells. The cells that were pre-treated with vitamin K2 exhibited remarkably less apoptosis when the cells were exposed to either hydrogen peroxide or β-amyloid. Further, vitamin K2 also decreased the levels of reactive oxygen species (ROS) in PC-12 cells that were exposed to hydrogen peroxide and β-amyloid. The levels of glutathione, an antioxidant, also increased when cells were pre-treated with 20 and 50 μM of vitamin K2. Hence, vitamin K2 pre-treatment reduced apoptosis signaling proteins (β-amyloid, caspase 3, *etc*.), attenuated levels of ROS, and increased levels of glutathione. In addition, it was reported that PC-12 cells when treated with hydrogen peroxide and β-amyloid increased phospho-p38 MAP kinase, PARP cleavage and Bax/Bcl-2 ratio; while pre-treatment with vitamin K2 resulted in decreased phosphorylation of p38 MAPK, PARP cleavage and mRNA level of Bax/Bcl-2. Inactivation of p38 MAPK pathway was identified as a mechanism for potential protective role of vitamin K2 in AD. Hence, the study confirms the protective role of vitamin K2 mediated by its antioxidant and anti-apoptotic properties (Hadipour et al., 2020).

**FIGURE 5:**
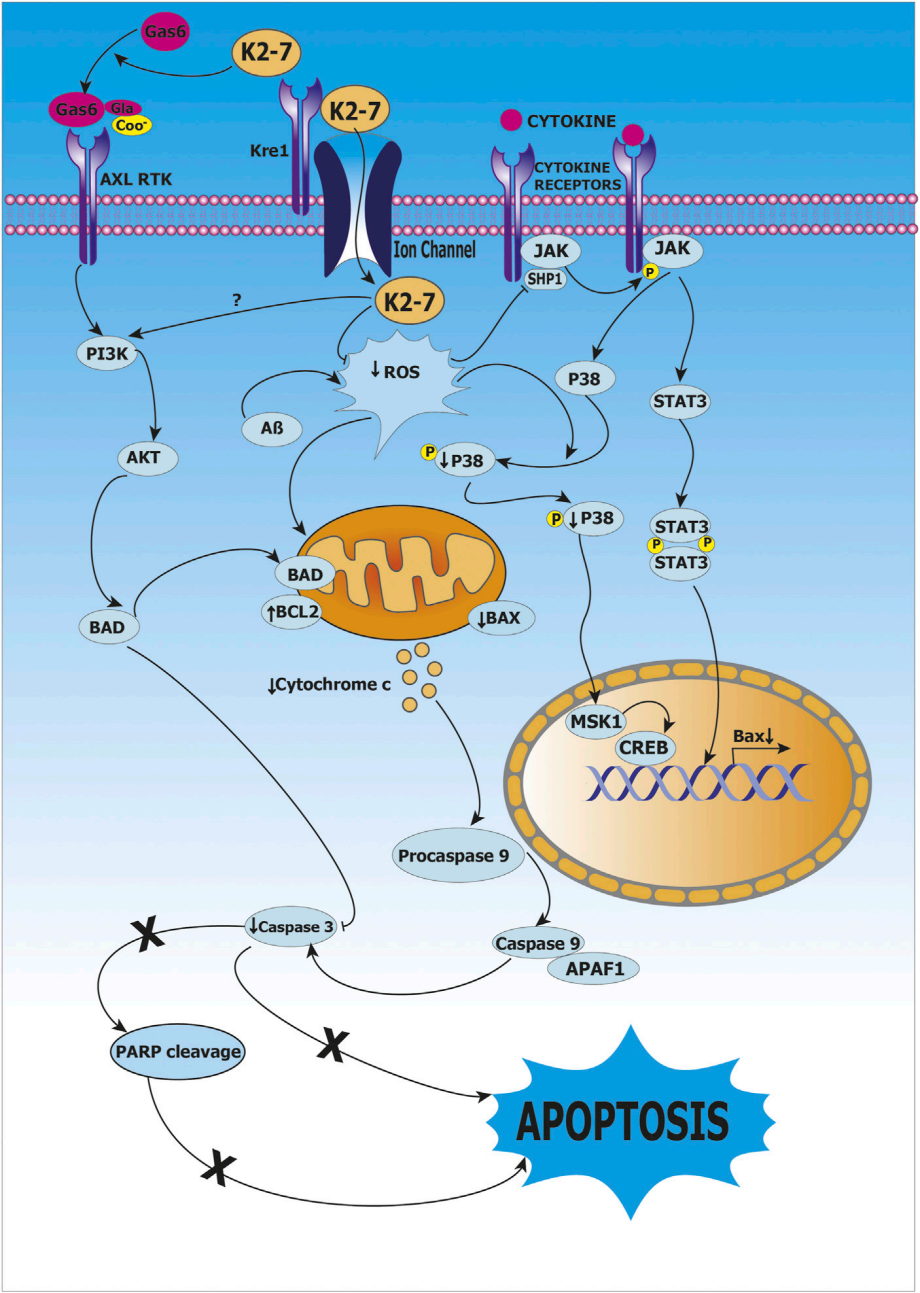
Role of vitamin K2-7 in Alzheimer’s disease. Vitamin K2-7 facilitates carboxylation of glutamate (Glu) residue present on growth arrest-specific protein 6 (Gas6) to γ-carboxyglutamate (Gla) leading to its activation, activated Gas6 binds to AXL receptor tyrosine kinase (AXL RTK) and initiates phosphoinositide 3-kinase/protein kinase B/BCL-2-associated death promoter protein (PI3K/AKT/BAD) signaling, BAD has an inhibitory effect on Caspase 3, this abrogates apoptosis and provides protection by K2-7 against β-amyloid (Aβ)-induced cytotoxicity. In cytosol, reactive oxygen species (ROS) activates Janus kinase-Signal transducer and activator of transcription protein (JAK-STAT) pathway resulting in transcription of BCL-2-associated X protein (BAX), BAX causes release of cytochrome c from mitochondria, cytochrome c leads to activation of Caspase 3 that results in apoptosis; vitamin K2-7 abrogates apoptosis by inhibiting Caspase 3.

Huang *et al.* studied the role of vitamin K2 as a protective agent using clones of rat astroglioma C6 cells that were transfected with C-terminal fragment of β-amyloid precursor protein (β-CTF/C6 cell model). Treatment of β-CTF/C6 cells with 0–10 µM vitamin K2 and 250 µg cumate for 72 h resulted in increased phosphorylation of Bcl-2-associated death promoter protein (Bad) and decrease in apoptosis mediated by caspase 3, this indicated that caspase 3-mediated apoptosis was inhibited by vitamin K2 *via* activation of protein Bad (Bcl-2 associated death promoter protein) which is an important mediator in the apoptotic pathway. Furthermore, it was reported that apoptosis mediated by β-amyloid is inhibited by activation of PI3K/Akt-signaling, as high levels of phosphorylated PI3K and Akt were observed after treatment of vitamin K2. Thus, PI3K/Akt/Bad-signaling pathway activation and inhibition of caspase-mediated apoptosis are the mechanisms underlying protective role of vitamin K2 as shown in (Huang et al., 2021) as shown in Figure 5.

Another factor that contributes to pathogenesis of Alzheimer’s disease is overactivated microglia. When microglia are overactivated, they trigger inflammatory cascades in the central nervous system. Disruption of microglial homeostasis leads to activation of neurotoxic astrocytes, synaptic loss and neuronal death resulting in inflammation and neurodegeneration (Spangenberg et al., 2016; Liddelow et al., 2017). Saputra *et al*. investigated vitamin K2 (MK-4) effect on activation of microglia and the underlying mechanism. In MG6 cells derived from mouse microglia exposed to lipopolysaccharide, different concentrations of vitamin K2 (0–10 µM) were used; wherein the upregulation of inflammatory cytokines like *IL-1β*, *IL-6* and *TNF-α* were highly suppressed at 10 μM at mRNA level. Vitamin K2 inhibited the nuclear translocation of NF-κB in MG6 cells induced by LPS, resulting in inhibition of NF-κB signaling. Further, phosphorylation of p65 was suppressed significantly due to the presence of vitamin K2 but the levels of TAK1 and IKKα/β were unaffected (Saputra et al., 2019).

## Pharmacometrics of Vitamin K2-7

### Clinical Pharmacokinetics of K2-7

ADME (absorption, distribution, metabolism, and excretion) kinetics are major factors that determine the safety and efficacy of K2-7. The absorption of K2-7 takes place from the lumen of ileum and jejunum. Absorption is rapid; K2-7 is incorporated into micelles or TAG-rich lipoproteins and doesn’t undergo any change during the process (Beulens et al., 2013). Micelles are packed into chylomicrons secreted by enterocytes; chylomicrons are then secreted out from within the intestinal villi *via* exocytosis into lymphatic system through thoracic duct and reach the circulatory system. Changes take place in the apoproteins of chylomicrons that contain K2-7, these changes help in their uptake *via* endocytosis in the bone osteoblasts, liver, and other tissues; endocytosis is mediated by lipoprotein receptors such as, low-density lipoprotein receptor (LDLR) and low-density receptor-related protein (LRP). Excretion of K2-7 involves shortening of isoprenoid side chains to form metabolites of carboxylic acid with 5-carbon side chains. Further, conjugation of these metabolites with glucuronic acid takes place and finally they are excreted in urine and bile (Shearer et al., 2012). A study reported that K2-7 and other long chain derivatives of vitamin K2 are readily available for the extra-hepatic tissues like vasculature and bone after redistribution to the circulatory system (Schurgers and Vermeer, 2002).

In a study conducted by Schurgers *et al.* (Schurgers et al., 2007), MK-7 (1500 mg/L) in an oil solution form was mixed with vitamin K1 resulting in a mixture containing 100 µg/ml of vitamin K1 and K2 (MK-7) each. 10 ml of this mixture was emulsified with 20 ml orange juice and administered to 15 volunteers between the ages of 25–35 years. The peak serum concentration (C_max_) of MK-7 was reported to be 17 µg/L and time required to achieve maximum concentration (T_max_) was reported to be 4 h. The decline in MK-7 serum concentration was slow between 8 and 9 h and followed a biphasic pattern. The half-life (t_1/2_) of MK-7 was reported to be 68 h. In a subsequent study, 10 volunteers received increasing doses (50, 100, 150, 200, 250, 300, and 500 µg each of the two vitamins) from a mixture of 100 µg/ml of vitamin K and MK-7. Both vitamins showed a linear dose response 4 h after mealtime. At 24 h after mealtime, the circulating concentration of MK-7 at 100 µg was reported to be approximately 1.5 nM (1 µg/L).

Sato *et al.* (Sato et al., 2012) investigated the bioavailability of menaquinone-7 in healthy Japanese women. Within 10 min post-breakfast consisting of 13–17 g fat, a single dose of MK-7 (420 μg) was given to five healthy female subjects. The single dose of MK-7 reached maximum serum concentration at 6 h after administration and the C_max_ was reported to be approximately 6.5 ng/ml. In a subsequent study, five healthy female subjects were administered with 60 μg of MK-7 daily after supper for 7 days. The serum level of MK-7 after consecutive administration for 7 days was reported to be 8.5 ng/ml thus leading to the conclusion that efficient absorption of nutritional dose of MK-7 is seen in humans with a significant rise in serum levels.

A study (Knapen et al., 2016) was performed in 107 volunteers in the Netherlands wherein postmenopausal women and healthy men (between 45 and 65 years of age) were subjected to a daily dose of *1*) MK-7-fortified yogurt (yogurt K), *2*) yogurt containing MK-7, magnesium, vitamin C and vitamin D3, fish oil and n-3 poly unsaturated fatty acids (n-3 PUFA) (yogurt Kplus) and *3*) soft gel capsules of MK-7, for 42 days. The MK-7 intake from either of the yogurts was reported to be 30.6 µg per serving while the soft gel capsules contained 58.3 ± 1.1 µg of MK-7. The participants were randomly assigned to receive either one soft gel capsule containing MK-7 or two servings of either yogurt K or yogurt Kplus daily for 42 days. The yogurt Kplus group was observed to have the highest plasma concentration during the initial 14 days of the treatment (2.75 ng/ml), while the plasma concentrations of yogurt K and capsules group in the same period were reported to be 2.5 and 2.25 ng/ml, respectively. Throughout the intervention of 42 days, the average plasma concentrations of the yogurt Kplus, yogurt K and capsule group were reported to be 2.29 ± 0.08 ng/ml, 2.17 ± 0.09 ng/ml, and 2.00 ± 0.09 ng/ml, respectively. Biphasic decline of MK-7 was observed during the 2-week washout period with a T_1/2_ of 3 and 8 days during the first phase and second phase, respectively. The plasma levels of dephospho-uncarboxylated matrix Gla protein (dp-ucMGP) was decreased to 485 ± 30 pmol/L in the yogurt Kplus group, 417 ± 33 pmol/L in the yogurt K group and 434 ± 31 pmol/L in the capsules group. Yogurt Kplus group was reported to have the largest improvement of MGP carboxylation, however this effect was majorly attributed to the response in women.

Moller *et al.* investigated the bioavailability of synthetic MK-7 and fermented MK-7 by conducting a single blinded two-way cross-over study. Synthetic or fermentation-derived MK-7 (180 µg) were administered orally as a single dose to nine patients and eight patients respectively. It was observed that the 90% confidence interval for the ratio of the AUC (0–72 h) values for synthetic and fermentation-derived MK-7 was 83–111, indicating bioequivalence. The 90% confidence interval for C_max_ ratio was 83–131 (Møller et al., 2017). Due to the widespread use of MK-7-containing supplements, the pharmacokinetics following single intake of MK-7 from various formulations was assessed in four parallel studies. Participants were administered either tablets or capsules containing 180 µg MK-7. It was observed that T_max_ of tablet (6 h) was slower as compared to capsules (2–4 h). This can be attributed due to the oily matrix of the capsules that released MK-7 more rapidly than the tablet powder matrix. It was also observed that all the formulations gave similar 24 h absorption profiles which indicated that the vehicle carrier does not affect the absorption of MK-7 (Mhj, 2014).

### Clinical Pharmacodynamics of K2-7

To estimate the effective dose of MK-7, Theuwissen *et al.* conducted a double blind, randomized, controlled study including 42 Dutch participants who were divided into seven groups and were administered with supplements (capsules) of placebo or different concentrations of MK-7 (10, 20, 45, 90, 180 or 360 µg) daily for 3 months. The plasma concentration range of MK-7 for the groups administered with 10, 20, 45, 90, 180 and 360 µg were reported to be 0–0.5, 0.4–2.1, 0–1.6, 0.4–4.6, 0.5–4.2, 1.1–6.5 and 4.1–10.6 ng/ml, respectively, thus indicating a direct correlation between plasma MK-7 concentration and MK-7 supplementation. Intake of 90 µg or more per day resulted in a significant increase in circulating concentrations as compared to placebo. Further, it was reported that MK-7 supplements at concentrations around recommended dietary allowance (RDA = 75 µg) (Theuwissen et al., 2012) enhanced carboxylation of matrix Gla protein (MGP) and osteocalcin (OC), while a decrease in the levels of circulating dephospho-uncarboxylayed MGP (dp-ucMGP) and uncarboxylated osteocalcin (ucOC) as well as in the ucOC:cOC ratio was observed. Following the highest intake of MK-7, the level of circulating cOC was significantly increased. No significant effects were observed on the circulating levels of osteocalcin and MGP at supplementation doses of MK-7 below RDA.

Knapen *et al.* (Knapen et al., 2018) conducted a randomized, placebo-controlled study including 214 postmenopausal women between the age group 0f 55–65 years of age wherein they received either 180 µg/day of vitamin K2 (MK-7) or placebo for 3 years. It was observed that MK-7 increased the levels of circulating osteocalcin (21.5%) as compared to placebo (2.6%) however there was no significant effect of MK-7 on body fat or fat distribution. In MK-7 group, levels of undercarboxylated osteocalcin (ucOC) were decreased by 50.1%, while in placebo group, no significant change was observed (+4.3%). In subjects that responded well with respect to osteocalcin carboxylation (good responders), MK-7 was reported to increase total and human molecular weight adiponectin, while abdominal fat mass and estimated visceral adipose tissue area were reported to be decreased as compared to poor responders as well as placebo. In good responders, the change in carboxylated osteocalcin was 1.66 ng/ml or 34.9% and in poor responders it was 0.38 ng/ml or 7.7%.

Biological effects of fermentation derived MK-7 (90 µg) and 3 doses of synthetic MK-7 (45, 90, and 180 µg) were compared in a randomized double-blinded parallel study. Placebo, 45 µg synthetic MK-7, 90 µg synthetic MK-7, 180 µg synthetic MK-7 or 90 µg fermentation-derived MK-7 were administered daily to the subjects for 43 days. Blood samples were collected for measurement of MK-7 as well as serum concentrations of cOC and ucOC. There was an increase in serum cOC concentration and a reduction in serum ucOC concentration after daily intake of the highest dose of synthetic MK-7 (180 µg) for 6 weeks. The fermentation-derived MK-7 group showed similar results. Therefore, it was concluded that the tested synthetic form of MK-7 is bioequivalent to fermentation-derived MK-7, exhibits vitamin K activity and can be well tolerated in healthy subjects (Møller et al., 2017).

A study was carried out by Aoun *et al.* to determine the level of dp-ucMGP, after treatment using MK-7 in haemodialysis patients. It is known that dp-ucMGP increases in vitamin K-deficient patients which can be associated with vascular calcification. Hence, 50 haemodialysis patients from Eastern Mediterranean cohort were administered 360 µg of MK-7 for 4 weeks. The results indicated 86% decrease of dp-ucMGP after 4 weeks of treatment; however, further studies are required to assess the change of vascular calcification after longer duration of treatment (Aoun et al., 2017). Also, a randomized dose-finding study was performed in 200 chronic hemodialysis patients. Patients were administered MK-7 with either 360, 720 or 1080 µg thrice weekly for 8 weeks. It was observed that decrease in dp-ucMGP was 17, 33 and 46% respectively indicating a dose dependent response by MK-7 supplementation. Few side-effects that were reported were mild and independent of the dose (Caluwe et al., 2014). Likewise, a randomized trial was carried out in 53 hemodialysis patients in which 45, 135 or 360 µg of MK-7 was administered daily for 6 weeks and compared with 50 healthy patients (control group). After 6 weeks of treatment, the plasma levels of the hemodialysis patients were assessed which indicated the level of dp-ucMGP and ucOC was 4.5- and 8.4-fold respectively higher than the control group. Forty-nine patients showed elevation in the protein induced by vitamin K absence II (PIVKA-II). It was also observed that MK-7 supplementation showed a dose- and time-dependent response in dp-ucMGP and ucOC and PIVKA-II levels (Westenfeld et al., 2012).

An experiment was performed by Brugè *et al.* to study the effect of K2-7 supplementation with extra-virgin olive oil on carboxylation of OC. Olive oil enriched with 45 or 90 µg of K2-7 was administered for 2 weeks in 12 healthy volunteers in Italy. The results demonstrated significant increase in cOC:ucOC ratio with 90 µg of K2-7 which improves bone mineralization; however, olive oil with 45 µg K2-7 did not show a biological effect. Hence, it was concluded that extra-virgin olive oil enriched with K2-7 can improve bone mineralization (Brugè et al., 2011). Dalmeijer *et al.* (Dalmeijer et al., 2012) conducted a randomized double-blind placebo-controlled study for 12 weeks to investigate the effect of K2-7 on carboxylation of matrix Gla protein (MGP). Sixty patients that were administered with 180 or 360 µg showed dose-dependent decrease in dp-ucMGP; however, no change was observed in dp-cMGP and total ucMGP level (Dalmeijer et al., 2012).

A randomized controlled trial was conducted by Sakak et al. (Rahimi Sakak et al., 2021) on 68 patients with type 2 diabetes on oral glucose-lowering therapy. Patients were either administered 360 μg of MK-7 or placebo daily for 12 weeks. No significant difference in the atherogenic status between the MK-7 or placebo group was observed. Hence, it was concluded that 360 μg of MK-7 supplementation for 12 weeks cannot improve the insulin resistant (IR)-related indexes of cardiovascular risk (Rahimi Sakak et al., 2021). Also, a randomized, double-blind placebo-controlled trial was performed to assess whether the supplementation of menaquinone-7 decreases vascular calcification in patients having type 2 diabetes and cardiovascular disease (CVD). Sixty-eight patients were randomly administered either with 360 μg daily of MK-7 or placebo for 6 months. Blood samples of the patients administered with MK-7 showed increased active calcification compared with placebo when measured with ^18^sodium fluoride positron emission tomography (^18^F-Na PET) activity; however, no effect was found in calcification mass on conventional computed tomography (CT) (Zwakenberg et al., 2019).

Inaba *et al.* investigated the effective minimum daily intake of MK-7 to improve osteocalcin γ-carboxylation. In postmenopausal Japanese women aged 50–69 years, a significant increase was observed in the ratio of osteocalcin/undercarboxylated osteocalcin and decrease in ucOC concentration when 100 and 200 µg was administered daily for 4 weeks of MK-7. Similar results were obtained in healthy males, and females aged 20–69 years in 100 µg daily intake of MK-7 for 12 weeks (Inaba et al., 2015). A randomized double-blind placebo-controlled trial was carried out in 334 early postmenopausal women. 360 µg of MK-7 in the form of natto capsules was administered daily for 1 year. Serum level showed significant improvement in cOC levels; however, there was no improvement in the bone mass density (BMD) in total hip, femoral neck, lumbar spine and total body. This suggested that there was no improvement in the bone loss rates after the 1 year treatment (Emaus et al., 2010).

The effect of MK-7 supplementation on the activity of vitamin K-dependent procoagulant factors was investigated by Ren et al. (Ren et al., 2021). Forty healthy volunteers were administered 90 µg of menaquinone-7 for 30 days; and prothrombin time (PT), activated partial thromboplastin time (APTT), thrombin time (TT), blood coagulation factors II, VII, IX and X activities and protein induced by vitamin K absence or antagonist-II (PIVKA-II) were measured in blood samples. The results indicated that supplementation of MK-7 at the recommended dose does not affect the vitamin K dependent coagulation factors (Ren et al., 2021). In a clinical study, Zhelyazkova-Savova *et al.* (Zhelyazkova-Savova et al., 2021) examined the effects of statin drug on vitamin K2 status and VKDPs. It was observed that the levels of uncarboxylated osteocalcin (ucOC) and ucOC:cOC ratio increased in patients that were administered with statin. In addition, statin inhibited activity of VKDPs and calcium accumulation was increased in arterial walls. A study conducted by Zhang et al., 2012. determined the synergistic effect of sorafenib and vitamin K2 on human hepatocellular carcinoma (HepG2) cells. *In vitro*, co-administration of 2.5 µM sorafenib with 1 µM vitamin K2 synergistically inhibited the HepG2 cell proliferation. *In vivo* study was carried out in nude mice by inoculation of EGFP-expressing HepG2 cells. Sorafenib (1.25 mg/kg body weight) and vitamin K2 (2 mg/kg body weight) were injected daily for 18 days. It was observed that sorafenib and vitamin K2 inhibited the tumor growth synergistically in combination therapy. In a study conducted by Hara et al. in rat aorta loop model, it was observed that when vitamin K2 (1.5, 14 and 145 mg/kg) and warfarin (0.80 mg/L) were administered simultaneously, the high dose of K2 (145 mg/kg) reduced the effect of warfarin on thrombosis suggesting on interaction between K2 and warfarin (Hara et al., 1999). A study was carried out by Theuwissen et al. to determine the effect of MK-7 supplementation on vitamin K antagonist therapy (VKA). Eighteen healthy volunteers were administered with increasing doses (10–20–45 µg) of MK-7 for 6 weeks after previous treatment with acenocoumarol for 4 weeks. The study reported that MK-7 supplementation as low as 10 µg should be avoided during VKA as it can significantly affect the anticoagulation sensitivity in few individuals (Theuwissen et al., 2013). Further, a study was designed to determine the effective role of MK-7 in the therapeutic management of rheumatoid arthritis (RA). Eighty-four patients undergoing RA treatment were either administered 100 µg dose of MK-7 capsules or kept naïve without changing any other medication for 3 months. The clinical and biochemical markers like ucOC, erythrocyte sedimentation rate (ESR), disease activity score assessing 28 joints with ESR (DAS28-ESR), C-reactive protein and matrix metalloproteinase (MMP-3) were assessed using patients’ serum. There was a significant decrease in clinical and biochemical markers such as ucOC, ESR and DAS28-ESR for moderate and good responders compared to non-responders. The results indicated that there was improvement in disease activity score of RA by changing the bone mineral metabolism when treated with MK-7 (Abdel-Rahman et al., 2015). Ozdemir et al. conducted a pilot study to investigate the therapeutic effect of dietary supplement of vitamin D (5 µg calcitriol) and vitamin K2 (50 µg MK-7) in 20 children on thalassemic osteopathy (TOSP). The serum samples collected after 6 and 12 months showed decrease in the ratio of ucOC to cOC; however, it was not significant (Ozdemir et al., 2013). Summeren et al. conducted a double blind randomized placebo-controlled trial to determine the effect of MK-7 supplementation on 55 pre-pubertal children aged between 6 and 10 years from Netherlands. 45 µg of MK-7 was administered daily for 8 weeks and compared with placebo group. The serum samples showed increase in MK-7 and decrease in circulating ucOC concentration (van Summeren et al., 2009).

### Clinical Trials on K2-7

Several clinical trials have been performed to determine the effect of vitamin K2-7 on various diseases and conditions that include bone disease, renal disease, diabetes mellitus type 2, thrombosis, *etc*. Studies also include the determination of effect of vitamin K2-7 on patients suffering from COVID-19. Various clinical trials for determination of the appropriate dose of vitamin K2 have been performed; also its bioavailability and efficacy have been investigated in a few trials. Further, in many trials, vitamin K2-7 is combined with vitamin D as an intervention to augment the beneficial effects. Vitamin K2-7 is taken in the form of drug (capsules) or included in dietary supplements such as dairy products (yoghurt, cheese, *etc*). These have been summarized in Table 3 and Table 4 for the better understanding of the reader.

**TABLE 3:** Clinical trials on vitamin K2-7 in the United States of America (Adapted from www.clinicaltrials.gov).

Clinical trial number	Status	Phase	Number of participants	Conditions or disease	Objective	Dose	Reference
NCT04770740	Recruiting	II	40	COVID-19	To study the effect of vitamin K2-7 in COVID	K2-7: 333 µg/day for 14 days	Dofferhoff, (2021)
						Placebo: 3 tablets/day for 14 days	
NCT02839044	Completed	NA	68	Arterial calcification, Type 2 Diabetes mellitus (T2DM)	To investigate the influence of vitamin K2	K2-7: 360 µg/day	Schouw, (2019)
						Placebo: Tablets daily	
NCT00165633	Terminated	II/III	540	Hepatocellular carcinoma	To detect the inhibitory effects on recurrence	Menatetrenone (E0167): 45/90 mg capsule thrice daily	Eisai Limited, (2008)
NCT00931437	Completed	NA	12	Healthy	To study the absorption from dairy	Vitamin K-rich dairy products	Maastricht University Medical Center, (2010a)
NCT03360435	Completed	NA	99	Post bariatric surgery patients	To study absorption of transdermal vitamins	Multivitamins	University of Florida, (2021)
NCT02917525	Unknown	II	44	Bicuspid aortic valve stenosis	To study the effect on calcium metabolism	360 µg vitamin K2 daily for 18 months	Maastricht University Medical Center, (2018a)
						Placebo for 18 months	
NCT00642551	Completed	NA	240	Bone loss Arteriosclerosis	To investigate long term beneficial effects	180 µg K2-7 daily for 3 years	Maastricht University Medical Center, (2012a)
						1 placebo capsule daily for 3 years	
NCT04641663	Recruiting	NA	70	Aging or age-related cognitive decline	To detect tolerability towards multi target dietary supplement (MTDS)	MTDS: Morning (five tabs), Evening (three tabs) and OMEGA (two softgels)	Boreham, (2021)
NCT04477811	Completed	II/III	40	Renal disease	Comparative study for vitamin K1 and K2	10 mg vitamin K1 thrice/week for 3 months	Farid, (2021)
						90 µg vitamin K2 per day orally	
NCT01873274	Completed	NA	107	Bioavailability	Comparative study for different delivery systems	Basic yogurt enriched with MK-7 (50 μg)	Maastricht University Medical Center, (2013)
						MK-7 containing capsule (50 μg)	
						Nutrient-enriched yogurt with MK-7	
NCT00858767	Completed	NA	69	Bioavailability	To examine arabic gum absorption	MK-7 from casein, arabic gum and linseed oil capsules	Maastricht University Medical Center, (2010b)
NCT00742768	Completed	NA	16	Healthy	Comparative study	Vitamin K2 in softgel Gelpell	Maastricht University Medical Center, (2018b)
NCT01638143	Completed	NA	24	Bioavailability	Bio-equivalence	Gnosis P-1000 capsule	Maastricht University Medical Center, (2012b)
						Gnosis M-1500 capsule	
						MenaQ7 M-1500 capsule	
NCT01638182	Completed	NA	81	Bone and vascular health	Bio-comparison	52 µg of vitamin K1	Maastricht University Medical Center, (2012c)
						75 µg of vitamin K2 for 3 months	
						1 placebo/day for 3 months	
NCT01194778	Completed	NA	82	Carboxylation level vitamin K-dependent proteins	To compare the efficacy	1 placebo sachet per day containing only sucrose for 12 weeks	Maastricht University Medical Center, (2010c)
						15 µg vitamin K1 per day for 12 weeks	
						15–45 µg (15 µg interval) vitamin K2 per day during 12 weeks for three groups	
NCT04382027	Completed	NA	20	Healthy	To study pharmacokinetics of omega-3 with vitamin K2	Omega-3 + vitamin K2 (monoacylglycerol form) Omega-3 + vitamin K2 (ethyl ester)	Plourde, (2020)
NCT03305536	Unknown	II	150	Aortic valve disease	To investigate decalcification of aortic valve	Vitamin K2 (1000 µg/day) + vitamin D3 (5000 IU/day)	Frangi, (2019)
NCT04780061	Recruiting	III	200	COVID-19	To use vitamin K2 as dietary supplement	Combination of vitamin K2 (30 µg) and vitamin D2 (3.125 µg)	The Canadian College of Naturopathic Medicine, (2021)
NCT00483431	Completed	NA	42	Vitamin K-status	Dose finding	MK7 10 µg (1 cap) + 3 placebo capsules	Maastricht University Medical Center, (2018c)
						MK7 20 µg (2 cap) + 2 placebo-capsules	
						MK7 45 µg (1 cap) + 3 placebo-capsules	
						MK7 90 µg (2 cap) + 2 placebo-capsules	
						MK7 180 µg (4 capsule)	
						MK7 360 µg (1 cap) + 3 placebo-capsules	
						All above doses taken daily for 12 weeks	
NCT04189796	Not yet recruiting	NA	64	Healthy women osteocalcin	Carlsberg vs camembert cheese	Oral intake of both cheese for 6 months	Larsen, (2019b)
NCT04041492	Completed	NA	100	Type 2 Diabetes mellitus, Bone Loss, Insulin resistance	To study effects of vitamin K2	Supplementation of vitamin K2 with vitamin D3 one capsule every 24 for 3 months	Ruiz, (2019)
NCT02976246	Completed	IV	123	Renal disease, Cardiovascular disease, Bone disease	To study effects of vitamin K2	1 tablet of MK-7 360 µg once daily	Zealand University Hospital, (2020)
						1 tablet of placebo once daily	
NCT04539418	Completed	IV	59	Renal disease	To determine effects of K2 on vascular calcification	Vitamin K2: thrice/week at the end of each dialysis session through the ultrafiltration membrane intravenously	Abúd, (2020)
NCT04010578	Not yet recruiting	NA	52	Coronary artery disease (CAD), Carotid artery disease	To study effects of vitamin K2 and vitamin D3 on CAD	400 µg of MK7 and 80 µg of vitamin D3/day	Mottaghy, (2021)
						Daily placebo for 3 months	
NCT04676958	Recruiting	NA	80	Inflammation, Oxidative Stress, Exercise, Strength, Recovery	To examine recovery of vitamin K2 from exercise	380 mg capsule/day micro-crystalline cellulose with 240 µg/day vitamin K2	Gray, (2021)
NCT01143831	Completed	NA	8	Thrombosis	Evaluation of proteins C and G; and factors II, VII, IX, X	Vitamin K2 in 5 mg capsules for 14 days	MD, (2012)
NCT01407601	Completed	III	53	Chronic kidney disease (CKD) Stage 5D, Hemodialysis	To activate matrix Gla protein (MGP)	45 μg, 135 and 360 µg of MK-7 daily prior to dialysis over 6 weeks	RWTH Aachen University, (2011)
NCT01101698	Unknown	IV	60	Kidney disease, Coronary artery calcification	To study the influence of vitamin K2	90 µg vitamin K2 with 10 µg vitamin D once daily for 9 months	Medical University of Lodz, (2010)
NCT04429035	Recruiting	NA	200	Aortic valve calcification and stenosis, Mitral annular calcification, Mitral valve calcification and stenosis	To slow down progress of calcification	300 µg vitamin K2 daily orally	Toutouzas, (2021)
						300 µg vitamin K2 and placebo daily orally	
NCT02404519	Unknown	NA	240	Vascular stiffness	To study effect of vitamin K2	180 µg K2-7 daily orally for 1 year	Knapen, (2018)
						1 Placebo tablet daily orally for 1 year	
NCT01922804	Unknown	NA	150	Metabolic bone disorder	To study effect on bone and glucose metabolism	Vitamin K2 375 µg/day for 3 years	University of Aarhus, (2016)
						1 Placebo tablet/day for 3 years	
NCT01870115	Completed	I	23	Osteopenia	To investigate effect of melatonin and micro-nutrients	2.5 mg melatonin, 225 mg strontium citrate, 1000 IU vitamin D3 and 30 µg vitamin K2	Duquesne University, (2018)
NCT01923012	Unknown	II	200	Carotid disease, Atherosclerosis	To determine effect of vitamin K2	Vitamin K2	Vidale, (2014)
NCT00290212	Completed	II	304	Perimenopausal bone loss	To investigate prevention of bone loss	Natto capsules + 360 µg vit K2/day	University Hospital of North Norway, (2008)
NCT04428606	Recruiting	NA	60	Pre-diabetes	To determine effect of synbiotics	Metabolic Rheostat™ (2 capsules 3× per day for 56 days)	Li, (2021)
						Butyrate Ultra (2 capsules 3× per day for 56 days)	
						Placebo (2 capsules 3× per day thrice for 56 days)	
NCT01675206	Completed	III	165	Vascular calcification	Dose defining study	360 μg, 720 and 1080 µg of vitamin K2 thrice weekly	Caluwe, (2012)
NCT03871322	Recruiting	NA	90	Fracture healing	To determine effects of vitamin D3 and K2 on fracture healing	Supplementation of vitamin D and K2	Medical University of Bialystok, (2021)
NCT04387019	Completed	-	90	Type 2 Diabetes mellitus	To study blood samples	Diagnostic test (samples from peripheral veins)	Helmy, (2021)
NCT02876354	Completed	IV	50	Vascular calcification	To evaluate risk factors	Menaquinone 360 µg /day for 4 weeks	Aoun, (2016)
NCT02970084	Completed	NA	60	Carotid plaques	ffect on calcium levels	Nutraceutical product based on vitamin K2, once a day (a tablet 800 mg) for 12 months	Tshomba, (2019)
NCT03799822	Completed	IV	132	Atrial fibrillation	Anti-coagulation	Rivaroxaban (10 mg) oral tablet + vitamin K2 (2000 µg) thrice weekly	Caluwe, (2020)
NCT00512928	Completed	NA	20	Healthy	To determine safety of vitamin K2 during anticoagulation	10–20 µg increasing to 45 µg for final week	Maastricht University, (2008)
NCT00548509	Completed	IV	131	Osteoporosis	To study the effect of K2 on bone turnover	Menatetrenone (Vitamin K2) therapy	Eisai Limited, (2018)
NCT01533441	Completed	II	50	Thrombosis	Intervention with antagonists of vitamin K (VKA)	4 months dairy product + treatment (vitamin K2 or placebo)	Danisco, (2014)
NCT02610933	Completed	IV	117	Vascular calcification	Replacement of VKA by Rivaroxaban + vitamin K2	Rivaroxaban (10 mg) tablet once daily and MK-7 (2000 µg) tablet thrice weekly for 18 months	Caluwe, (2019)
NCT03243890	Completed	NA	389	Aortic valve stenosis	Decalcification	720 µg/day MK7 and vitamin D (25 µg/day)	Diederichsen, (2021)
NCT01002157	Unknown	NA	180	Coronary artery disease (CAD)	Effect on progression of coronary artery calcification	Vitamin K2	Maastricht University Medical Center, (2018d)
						Placebo control (containing no vitamin K2)	
NCT01928134	Unknown	NA	60	Healthy	Efficacy of vitamin K2	Vitamin K2 + Vitamin D3 + calcium carbonate (CaCO_3_)	Su, (2013)
NCT04145492	Unknown	II/III	60	End stage renal disease (vascular calcification)	Effect of vitamin K2	90 µg of vitamin K2-7 daily + standard therapy for 4 months	Borolossy, (2019)
						Vitamin K2-7 (90 µg) + 10 µg of vitamin inactive vitamin D daily + standard therapy for 4 months	
NCT00402974	Completed	NA	55	Healthy	Effect on osteocalcin carboxylation in children	45 µg vitamin K2 for 8 weeks	UMC Utrecht, (2008)
NCT02870829	Active, not recruiting	II	178	Systemic and arterial stiffness complication of hemodialysis	Reduction of vascular calcification	Oral supplement of MK7 post dialysis thrice/week	Peixin, (2020)
NCT02366481	Active, not recruiting	NA	30	Obesity, Insulin resistance, Insulin sensitivity, β-cell dysfunction, Pre-diabetes	Effect on glucose metabolism in adults	Low dose: 1 vitamin K2 (90 µg) and 1 placebo softgel capsule daily for 8 weeks	Pollock, (2019a)
						High dose: 2 vitamin K2 (90 µg) daily for 8 weeks	
NCT01972113	Recruiting	NA	30	Obesity, Insulin resistance, Insulin sensitivity, β-cell dysfunction, Pre-diabetes	Effect on glucose metabolism in children	Low dose: 1 vitamin K2 (45 µg) and 1 placebo softgel capsule daily for 8 weeks	Pollock, (2019b)
						High dose: 1 vitamin K2 (90 µg) daily for 8 weeks	
NCT04900610	Not yet recruiting	NA	120	Peritoneal dialysis	Effect on arterial stiffness and cardiovascular events	Vitamin K2 1 mg/day orally	Vasileios, (2021)
NCT03311321	Recruiting	NA	60	Cardiovascular disease (CVD), Chronic kidney disease (Stage 3, 4 and 5)	Slow down progression of CVD risk in hemodialysis patients	4 vitamin K2 (90 µg) softgel capsules daily for 8 weeks	Pollock, (2019c)
						4 placebo softgel capsules daily for 8 weeks (without vitamin K2)	
NCT02959762	Active, not recruiting	NA	30	Insulin resistance, Obesity in diabetes, Nutritional and metabolic diseases, Hyperlipidemia, Hyperglycemia	Slow down progression of Dyslipidemia and diabetes risk in children	Placebo control for 8 weeks	Pollock, (2019d)
						Low dose: 1 vitamin K2 (45 µg) and 1 placebo softgel capsule daily for 8 weeks	
						High dose: 2 vitamin K2 (45 µg) daily for 8 weeks	
NCT02517580	Completed	II	60	Arterial stiffness	Effect on arterial stiffness in the renal transplant population	Vitamin K2 (360 µg/day) once daily for 8 weeks	Ph.D, (2017)
NCT03493087	Completed	NA	33	Renal Insufficiency, Chronic	Why hemodialysis patients have a low vitamin K status and how to improve it	MK-7 360 µg tablet /day for 6 weeks	NCT03493087. (2019)
						Diet rich in vitamin K for 6 weeks	
NCT04669782	Active, not recruiting	NA	123	Bone Metabolism Disorder, Aging Disorder and, Osteoporosis	Effect on bone and skeletal muscle, and energy metabolism in patients with severe osteoporosis	MK-7 (375 µg/day) + Teriparatide	University of Milano Bicocca, (2020)
						Teriparatide will be administered as standard	
NCT04285450	Recruiting	IV	99	Diabetes Mellitus, Type 2	Effect on the glycemic control, insulin sensitivity and lipid profile	Vitamin K 1 mg	Mohamed, (2020)
						Placebo	
NCT03897660	Completed	NA	22	Healthy	Better bioavailability of Omega-3 and Vitamin K2	Omega-3 + vitamin K2 (TG form of omega-3)	Plourde, (2020)
						Omega-3 + vitamin K2 (EE form of omega-3)	
						Omega-3 + vitamin K2 [MaxSimil (MAG form of omega-3)]	
NCT03813550	Unknown	NA	20	Pseudoxanthoma Elasticum (PXE)	Association between gut microbiota composition, plasma levels of vitamin K and severity of clinical manifestations in PXE patients	Not Applicable	University Hospital, Angers, (2019)
NCT04188080	Unknown	NA	12	Maintenance dose, Osteocalcin, Vitamin K deficiency	To estimate the maintenance of daily dose of Jarlsberg cheese	Maintaining daily dose of Jarlsberg cheese	Larsen, (2019a)
NCT04612088	Active, not recruiting	NA	34	Bariatric patients	To promote multivitamin adherence	Bariatric Multivitamin (two tablets once a day for 3 months)	Sanchez, (2021)

**TABLE 4:** Other global clinical trials on vitamin K2-7.

Clinical trial number	Status	Phase	Number of participants	Conditions or disease	Objective	Dose	Reference
ISRCTN18436190	Completed	NA	95	Anteroposterior sway	To improve balance and reduce the risk of falls in older people	Oral vitamin K2 (200 µg or 400 µg once daily for 1 year)	ISRCTN - ISRCTN 18436190. (2018)
ISRCTN21444964	Completed	NA	159	Chronic kidney disease stages 3B and 4	To study effect on pulse wave velocity	400 µg once daily oral vitamin K2 (MK7 subtype) vs matching placebo	ISRCTN - ISRCTN21444964. (2015)
ISRCTN93213492	Completed	NA	80	Vascular disease	To study effect of vitamin K supplementation on markers of vascular health in older people	Vitamin K2 (MK7 subtype) 100 µg per day or placebo	ISRCTN - ISRCTN93213492. (2012)
NL5147	Unknown	NA	NA	Diabetes patients, arterial calcification, bone metabolism	To reduce ongoing calcification and significantly reduce dephosphorylated-uncarboxylated mat dp-ucMGP	Vitamin K2 (menaquinone-7)	NTR NL5147. (2016)
NL1338	Open for patient inclusion	NA	140	Postmenopausal women	To reduce osteoclast activity and increase carboxylation of osteocalcin and MGP	Dairy product low vitamin K	NTR NL1338. (2009)
						Dairy product + 100 µg vitamin K1	
						Dairy product + 100 µg vitamin K2	
NL1876	Planned	NA	60	CVD, Atherosclerosis	Vitamin K2 can increase the carboxylation of the matrix Gla protein (MGP)	Two groups will receive a daily dose of 180 or 360 μg vitamin K2 respectively for 12 weeks	NTR NL 1876. (2011)
						Placebo group: 1 capsule daily	
NL9013	Planned	NA	20	Cardiovascular disease	To reduce the risk of coronary calcification	1 sachet of probiotics producing vitamin K2 for 12 weeks	NTR NL9013. (2021)
						Placebo	
NL7296	Planned	NA	20	Vitamin K deficiency, Cardiovascular risk	To improvise vitamin K status	A daily sachet of 4 gm dried multispecies probiotics or placebo	NTR NL7296. (2020)
DRKS00016046	Completed	NA	30	Healthy	To study efficacy of topical application of fat-soluble vitamins in liposomes	Vit A (780 IU) + Vit D (1500 IU) + Vit E (1.38 IU) + Vit K2 (66 µg), Vit A (1560 IU) + Vit D (3000 IU) + Vit E (2.77 IU), Vit K2 (133 µg) Vit A (3120 IU), Vit D (6000 IU), Vit E (5.54 IU), Vit K2 (265 µg) for 3 weeks (blood draw in the last 3 days of the intervention)	DRKS00016046. (2018)
jRCTs051180115	Recruiting	NA	200	Hepatocellular carcinoma (HCC)	TACE (transcatheter arterial chemoembolization) + vitamin K treatment	Daily dosing of vitamin K2 (45 mg) for 1 day before TACE to 28 days after	JRCTs051180115. (2019)
UMIN000033832	Pending	NA	15	Healthy adult	To study osteogenesis by intake of vitamin K2-rich natto	Natto	UMIN000033832. (2018)
UMIN000030521	Complete: follow-up continuing	NA	10	Warfarin treated patients	To evaluate PT-INR (Prothrombin Time- International Normalized Ratio)	Warfarin-treated patients who eat low vitamin K2 Natto for 7 days. When 10 g is acceptable, same patients will take 20 g Natto	UMIN000030521. (2018)
IRCT20190824044592N1	Recruitment complete	III	68	Type 2 diabetes	To study effect on glycemic control and lipid profile	Vitamin K2 (180 µg capsule twice a day for 12 weeks)	IRCT. (2020)
						Placebo capsule twice daily for 12 weeks	
IRCT201506185623N45	Recruitment complete	III	66	Type 2 diabetes and CVD	To determine effects of combined calcium, vitamins D and K supplementation	Vit D (200 IU) + Vit K2 (90 µg) + calcium (500 mg) twice a day for 12 weeks	IRCT. (2019a)
						Placebo twice a day for 12 weeks	
IRCT20100123003140N22	Recruitment complete	III	46	Type 2 Diabetes	To study effect on metabolic, nutritional, inflammatory, and matrix γ-carboxyglutamate protein	Menaquinone supplement (200 g/day)	IRCT. (2019b)
						Placebo (for 3 months)	
IRCT2017092836204N2	Recruitment complete	II / III	84	Polycystic ovary syndrome (PCOS)	To investigate effect on body mass index (BMI), waist circumference (WC), fasting blood sugar (FBS), lipid profile, endocrine markers and MDA	1 capsule of menaquinone per day	IRCT. (2022)
						Placebo	
IRCT20170916036204N5	Recruitment complete	NA	84	Polycystic ovary syndrome (PCOS)	Effect on depression status, fasting glucose and serum vitamin K level in patients with PCOS	1 menaquinone capsule (90 µg) per day	IRCT. (2021)
						Placebo	
IRCT201509015623N51	Recruitment complete	III	60	PCOS	Effects of vitamins D, K and calcium supplementation on metabolic profiles in women with PCOS	Vitamin D + Vitamin K + Calcium	IRCT. (2018)
						Placebo	
ACTRN12619000170123	Completed	NA	125	Mild hypercholesterolemia cardiovascular diseases	Effect on lipid profile	BruMeChol^™^ twice/day for 12 weeks	ACTRN12619000170123. (2020)
ACTRN12619000102178	Completed	NA	88	Hypercholesterolemia Mixed dyslipidemia	Effect on lipid profile, oxidative stress and inflammation	Octacosanol (20 mg) + Vitamin K2 (45 µg)	(ACTRN12619000102178. (2021))
2019–004906–88	Ongoing	III	40	Cardiovascular diseases	Effect on vascular calcification	Vitamin K2 MK-7 capsule (360 µg)	Clinical Trials Register. (2019)
CTRI/2016/11/007499	Completed	NA	100	Type 2 diabetes mellitus Vitamin B12 deficiency	Effect of Vitamin K2 supplementation	Vitamin K2-7 capsule (100 µg) twice a day for 8 weeks	CTRI/2016/11/007499. (2017)
CTRI/2013/09/003998	Completed	NA	20	Idiopathic muscle cramps	Effect of Vitamin K2 supplementation in Idiopathic muscle cramps	Vitamin K2-7 capsule (100 µg) twice a day for 3 months	CTRI/2013/09/003998. (2021)
CTRI/2012/08/002930	Completed	NA	30	Megaloblastic Anaemia, Type 2 diabetes mellitus	Effect of Vitamin K2 supplementation	Vitamin K2-7 capsule (100 µg) twice a day for 8 weeks	CTRI/2012/08/002930. (2017)
CTRI/2017/09/009660	Completed	NA	16	Healthy	Bioavailability study of vitamin K2	Vitamin K2-7 (1000 µg)	CTRI/2017/09/009660. (2017)
CTRI/2017/06/008925	Completed	NA	60	Vitamin B12 deficiency, T2DM	Effect of Vitamin K2 supplementation	Vitamin K2-7 capsule (100 µg) twice a day for 8 weeks	CTRI/2017/06/008925. (2017)
						Placebo	
CTRI/2018/05/014246	Completed	NA	80	Type 2 Diabetes mellitus with Hypertension	Evaluation of plasma levels of Vitamin K2-7	NA	CTRI/2018/05/014246. (2021)
CTRI/2019/03/018278	Completed	NA	50	Healthy	Evaluation of plasma levels of Vitamin K2-7	NA	CTRI/2019/03/018278. (2019)
CTRI/2019/06/019548	Completed	NA	20	Type 2 diabetes mellitus and Vitamin B12 deficiency	Plasma levels of Vitamin K2 upon oral supplementation	Vitamin K2-7 capsule (100 µg) twice a day for 8 weeks	CTRI/2019/06/019548. (2020)
						Placebo	
CTRI/2019/12/022361	Completed	NA	15	Healthy	Pharmacokinetics of Vitamin K2-7	Vitamin K2-7 capsule (350 µg) for 21 days	CTRI/2019/12/022361. (2021)

**(**Adapted from ISRCTN, International Standard randomized Controlled Trial Number (www.isrctn.com); NL, Netherlands Trial Register (www.trialregister.nl); DRKS, German Clinical Trials Register (www.drks.de); jRCT, Japanese Registry for Clinical Trials (https://jrct.niph.go.jp/) UMIN, University hospital Medical Information Network (UMIN) Clinical Trials Registry (www.umin.ac.jp); IRCT, Iranian Registry of Clinical Trials (www.irct.ir); ACTRN, Australian Clinical Trials Registration Number (www.australianclinicaltrials.gov.au); EUCTR, European Union Clinical Trials Register (www.clinicaltrialsregister.eu); CTRI, Clinical Trials Registry- India (www.ctri.nic.in)**)**.

## Contraindications to Vitamin K2-7

There are no severe adverse effects due to the supplementation of vitamin K2-7. However, high doses of vitamin K2 can cause allergic reactions (Korah et al., 2017). In a study conducted to find the effect of K2-7 on warfarin on thrombotic rate using a rat aorta model, it was observed that high dose of vitamin K2-7 (145 mg/kg) reduced the effect of warfarin (0.80 mg/L) on thrombosis (Hara et al., 1999). There are no other known major contraindications to K2-7 intake as of current knowledge.

## Conclusions

Vitamin K2-7 or menaquinone-7, a naturally occurring form of vitamin K2, is easily absorbed and readily bioavailable compared to other forms. Supplementation of the diet with K2-7 has health-beneficial effects in various diseases including osteoporosis, cardiovascular diseases, cancer, diabetes and related complications, and neurodegenerative diseases. The molecular underpinnings of these beneficial effects involve complex regulatory crosstalk between important signal transduction cascades in the cellular milieu. It can also potentially involve the interplay of noncoding RNAs such as miRNAs and lncRNAs which merit further investigation. Clinical evidence from our research group has unequivocally demonstrated the utility of K2-7 dietary supplementation for peripheral neuropathy. Taken together, the health-beneficial effects of K2-7 in various diseases underscore the need for supplementation of K2-7 in the global diet as evidenced by clinical and molecular data. Some of the trials on vitamin K2-7 that enrolled people with chronic diseases such as diabetes were limited to 8–12 weeks duration. This underscores the lack of adequately-designed intervention trials for K2-7 that can truly capture the real-world evidence-based scenario in clinical therapeutics. Nevertheless, these trials, despite their limitations, provide a proof-of-concept for the utility of K2-7 intervention, which must necessarily be followed up with larger, better-designed and suitably longer duration trials.

## Glossary

AD: Alzheimer’s disease
AKT: Protein kinase B/PKB
AMPK: AMP-activated protein kinase
Bad: Bcl-2-associated death promoter protein
Bax: Bcl-2-associated X protein
Bcl-2: B-cell lymphoma 2
BMP: Bone morphogenetic protein
CDK: Cyclin-dependent kinases
CKD: Chronic kidney disease
CVD: Cardiovascular disease
ERK: Extracellular signal-regulated kinase
Gas6: Growth arrest-specific protein 6
HCC: Hepatocellular carcinoma
HepPPS: Heptaprenyl pyrophosphatase synthetase
IKK-α: Inhibitor of nuclear factor kappa-B kinase subunit alpha
JNK: c-Jun N-terminal kinase
MAPK: Mitogen-activated protein kinase
MGP: Matrix Gla protein
MK: Menaquinone
NF-κB: Nuclear factor kappa light chain enhancer of activated B cells
OC: Osteocalcin
OPG: Osteoprotegerin
PARP: Poly-ADP ribose polymerase
PI3K: Phosphoinositide 3-kinase
PPAR-α: Peroxisome proliferator-activated receptor
pRb: Retinoblastoma protein
RANKL/RANK: Receptor activator for nuclear factor kappa ligand/receptor
ROS: Reactive oxygen species
SOCS: Suppressor of cytokine signaling
STAT5B: Signal transducer and activator of transcription 5B
TAG: Triacylglycerol
TAK: Transforming growth factor-β-activated kinase 1
TAM: Tyro3, Axl, and Mer family
VKA: Antagonist of vitamin K
VSMC: Vascular smooth muscle cells
